# Calcium Ion Induced Structural Changes Promote Dimerization of Secretagogin, Which Is Required for Its Insulin Secretory Function

**DOI:** 10.1038/s41598-017-07072-4

**Published:** 2017-08-01

**Authors:** Jae-Jin Lee, Seo-Yun Yang, Jimin Park, James E. Ferrell, Dong-Hae Shin, Kong-Joo Lee

**Affiliations:** 1 Graduate School of Pharmaceutical Sciences, College of Pharmacy, Ewha Womans University, Seoul, 120-750 Korea; 2 Department of Chemical and Systems Biology, Stanford University School of Medicine, Stanford, CA 94305-5174 USA

## Abstract

Secretagogin (SCGN), a hexa EF-hand calcium binding protein, plays key roles in insulin secretion in pancreatic β-cells. It is not yet understood how the binding of Ca^2+^ to human SCGN (hSCGN) promotes secretion. Here we have addressed this question, using mass spectrometry combined with a disulfide searching algorithm DBond. We found that the binding of Ca^2+^ to hSCGN promotes the dimerization of hSCGN via the formation of a Cys193-Cys193 disulfide bond. Hydrogen/deuterium exchange mass spectrometry (HDX-MS) and molecular dynamics studies revealed that Ca^2+^ binding to the EF-hands of hSCGN induces significant structural changes that affect the solvent exposure of N-terminal region, and hence the redox sensitivity of the Cys193 residue. These redox sensitivity changes were confirmed using biotinylated methyl-3-nitro-4-(piperidin-1-ylsulfonyl) benzoate (NPSB-B), a chemical probe that specifically labels reactive cysteine sulfhydryls. Furthermore, we found that wild type hSCGN overexpression promotes insulin secretion in pancreatic β cells, while C193S-hSCGN inhibits it. These findings suggest that insulin secretion in pancreatic cells is regulated by Ca^2+^ and ROS signaling through Ca^2+^-induced structural changes promoting dimerization of hSCGN.

## Introduction

Secretagogin (SCGN) is a Ca^2+^ sensor protein that possesses six EF-hand helix-loop-helix calcium binding motifs. *Scgn*, originally cloned from pancreatic β cells^1^, is expressed selectively in pancreatic β cells and neuroendocrine cells^1^, as well as at distinct neurosecretory loci in the brain^2, 3^. SCGN controls stress hormone release^2^ and olfactory neuron differentiation in Alzheimer’s disease^4^. Recently, we reported that SCGN also plays crucial roles in insulin secretion in pancreatic β cells by interacting with vesicle fusion and trafficking proteins^1, 5^ or with actin cytoskeleton^6^. We also showed that SCGN regulates focal adhesion by regulating the actin cytoskeleton during insulin secretion. SCGN physically interacts with actin and this interaction is increased by H_2_O_2_ and glucose stimulation, indicating that Ca^2+^ and reactive oxygen species (ROS) regulate the interaction between SCGN and actin. ROS responsive insulin secretion was diminished by silencing SCGN which further indicates that SCGN mediates Ca^2+^ and ROS signaling in insulin secretion.

In pancreatic β cells, intracellular Ca^2+^ and ROS are known to play key roles in glucose-stimulated insulin secretion (GSIS) by regulating intracellular signaling and nutrient sensing, coupling glucose metabolism to insulin secretion^7–11^. Subtle changes in Ca^2+^ signaling affect β-cell physiology and the pathogenesis of diabetes. Ca^2+^ signaling is mainly mediated by Ca^2+^ binding proteins, one of which is SCGN^12^. SCGN has a low Ca^2+^ affinity and functions as a Ca^2+^ sensor protein, controling protein-protein interactions and cellular signaling via conformational changes^13, 14^. ROS induce oxidative modifications, including reversible disulfide bonds, and structural and functional changes that modulate the signaling activity of proteins^15^. Dimerization of re-folded recombinant SCGN, possibly due to oxidation, has been reported^16^, raising the possibility of physiological redox regulation of SCGN. Recently it was reported that SCGN is a redox sensitive Ca^2+^ sensor, and that this dimerization and Ca^2+^ binding increase the stability of SCGN^17, 18^. However, the structural regulation and functional implication of Ca^2+^ binding and dimer formation of SCGN remain to be elucidated.

An x-ray structure of the calcium-free *Danio rerio* SCGN monomer (apo-SCGN) has been determined, but not that of SCGN in the presence of Ca^2+^ ^19^. Hydrogen/deuterium exchange monitored by mass spectrometry (HDX-MS) provides a complementary method for investigating structural change to x-ray crystallography. In previous work employing HDX-MS, we identified structural changes in the NDP kinase protein (Nm23-H1) caused by stepwise oxidation^20^. Here we report on HDX-MS studies on the structural changes in human SCGN (hSCGN) to see how its Ca^2+^ binding might affect its structure and impact upon insulin secretion. We validated these findings by molecular modeling of Ca^2+^ bound hSCGN. Our findings indicate that Ca^2+^ binding promotes the formation of stable dimers of hSCGN in the presence of ROS, and suggest that this dimerization is necessary for SCGN to promote insulin secretion.

## Methods

### Reagents and plasmids

ViaFECT transfection reagent: Promega (Madison, WC, USA), Effectene transfection reagent: QIAGEN (Valencia, CA, USA), Insulin ELISA kit: ALPCO (Windham, NH, USA), BCA protein assay: Thermo Scientific (Rockford, IL, USA). For expression in mammalian cells, the constructs encoding pcDNA3.1 Human SCGN WT, mutants C193S, C253S, C269S and ΔEF1 (a.a.: Δ1-52) were generated by cloning and mutation using the pcDNA3.1 myc his (−) A vector and an SCGN cDNA clone obtained from human cDNA library as a template. All plasmid constructs were confirmed by DNA sequencing.

### Constructs and protein purification

pGEX-1λT-h*Scgn* was provided from Dr. Ludwig Wagner (Univ. of Vienna, Austria). Mutant hSCGN C193S, C253S and C269S were generated using the Quik-Change^®^II site-directed mutagenesis kit (Agilent Technologies) according to the manufacturer’s protocol using pGEX-1λT-*Scgn* as a template. The mutagenesis oligonucleotides used were the following: for mutation C193S, sense primer 5′-TTCTCCAATTTAAAATGGATGCTAGTTCTACTGAAGAAAGGAAAAGG-3′, antisense primer 5′-CCTTTTCCTTTCTTCAGTAGAACTAGCATCCATTTTAAATTGGAGAA-3′; for mutation C253S, sense primer 5′-ATTCTCCTGCGTCACAGCGACGTGAACAAGG-3′, and antisense primer 5′-CCTTGTTCACGTCGCTGTGACGCAGGAGAAT-3′; for mutation C269S, sense primer 5′-AGTCTGAGCTGGCTTTGAGTCTTGGGCTGAAAATC-3′, and antisense primer 5′-GATTTTCAGCCCAAGAC TCAAAGCCAGCTCAGACT-3′ (mutated codons are underlined). Construct integrity was validated by DNA sequencing.

A pGEX-1λT plasmid carrying the human *Scgn* gene was expressed in BL21 (DE3) *E. coli* cells. The bacteria were cultured in LB medium at 37 °C, and recombinant fusion protein production was induced with 0.5 mM isopropyl-β-D-thiogalactopyranoside (IPTG). After 4 h of additional incubation at 37°C, the cells were harvested and lysed by vortexing with lysozyme containing hSCGN purification buffer (lysozyme, protease inhibitor, 5 mM DTT, 10 mM EDTA in PBS) on ice. The soluble protein fraction was recovered by centrifugation at 60,000 g for 1 h at 4°C. The GST-fused recombinant proteins in the supernatant were purified by chromatography on a glutathione-agarose column followed by washing, and equilibration by hSCGN elution buffer (140 mM NaCl, 10 mM HEPES, pH 7.4). To cleave hSCGN from the GST-hSCGN, the beads were incubated overnight at room temperature with thrombin in SCGN elution buffer. After 24 h, the purified hSCGN was eluted and quantity was measured.

### Cell culture and transfection

HeLa cells were purchased from ATCC and cultured in EMEM supplemented with 10% fetal bovine serum (FBS), 100 μg/mL streptomycin, 100 units/mL penicillin G at 37 °C in an atmosphere of 5% CO_2_-95% air. NIT-1 insulinoma cells were maintained in 5.6 mM glucose DMEM supplemented with FBS and antibiotics as above. For transient overexpression of specific proteins in HeLa cells, cells were transfected using Effectene and analyzed at 24 h post transfection. For NIT-1 cells, the cells were transfected using ViaFECT and analyzed at 48 h post transfection according to the manufacturer’s recommendations.

### Disulfide analysis of SCGN using nanoUPLC-ESI-q-TOF tandem MS and DBonds algorithm

The peptides resulting from trypsin-digested protein in non-reducing gel were resuspended in 10% acetonitrile containing 0.1% formic acid and analyzed using nanoAcquity™ UPLC™/ESI/MS (SYNAPT™ HDMS™, Waters Co. UK) as described previously^6^. Tandem MS (MS/MS) spectra were matched against amino acid sequences in the SwissProt human database (version 57.8., 20401 entries) using DBond (https://prix.hanyang.ac.kr).

### NPSB-B label

Methods for the labeling of reactive cysteine residues by NPSB-B were previously reported^21^. Briefly 2 *μ*g of recombinant hSCGN was pre-incubated with/without Ca^2+^ at room temperature for 15 min followed by incubation with NPSB-B (final concentration 1 mM) at room temperature for 2 h. Proteins were separated by 12% SDS PAGE and transferred to PVDF membranes. Labeled proteins were detected by streptavidin-HRP. The amount of total loaded protein was detected by Coomassie staining.

### Hydrogen/deuterium exchange mass spectrometry (HDX-MS)

1 µL hSCGN (1 mg/mL) was diluted 19-fold with labeling buffer (10 mM HEPES in D_2_O, pH 7.4) and incubated at 25°C for10, 60, 300, 1800, or 10800 s. The deuterium labeling reaction was quenched by 2.5 mM tris (2-carboxyethyl) phosphine (TCEP), formic acid, pH 2.3. For protein digestion, 1 μg of porcine pepsin was added to each quenched protein sample and incubated on ice for 3 min before injection. Peptic peptides were desalted on C18 trap column cartridge (Waters, UK) and gradient eluted from 8% CH_3_CN to 40% CH_3_CN, 0.1% formic acid on 100 µm i.d. × 100 mm analytical column, 1.7 µm particle size, BEH130 C_18_, (Waters, UK) for 7 min. The trap, analytical column and all tubing were immersed in an ice bath to minimize deuterium back-exchange. Gradient chromatography was performed at a flow rate 0.5 μL/min and was sprayed on line to a nanoAcquityTM/ESI/MS (SYNAPT^TM^ HDMS^TM^) (Waters, UK). The extent of deuterium incorporation was monitored by the increase in mass of the isotope distribution for each identified peptide, and calculated using Microsoft Excel. The theoretical maximum deuterium incorporation value was calculated for each peptide based on the number of exchangeable amides^20^. Each experiment was performed in triplicate. Deuterated peptides were calculated by DynamX v3.

### Model building and molecular dynamics (MD) simulation

The MODELLER 9.9 program (http://salilab.org/modeller/) was used to generate homology models of hSCGN. The 3D structure of SCGN from *Danio Rerio* (PDB ID: 2BE4)^19^ which has high sequence identity to hSCGN (~70%) was used as a template. A Ca^2+^-bound hSCGN structure was manually constructed with COOT^22^. Six Ca^2+^ ions were added to each potent EF-hand loop (Supplementary Table S2)^23^ and the complex was energy minimized in SYBYL-X 2.1. The modeled structures of hSCGN and Ca^2+^-bound hSCGN were used for the MD simulations. Each coordinate was solvated in water molecules and ionized with NaCl to a final equivalent concentration of 150 mM in order to neutralize charge. All MD simulations were performed with the NAMD 2.9 package using the CHARMM27 force field for proteins with the CMAP correction and the TIP3P model for water^24^. Each system was energy-minimized in two phases totaling 10,000 minimization steps. Water molecules and ions were relaxed in the 5,000 minimization steps and then protein was relaxed in the next 5,000 minimization steps. The minimized systems were heated to 300 K gradually and equilibrated at 300 K. After equilibration, MD simulations were performed for 40 ns in an NPT ensemble using 2 fs integration step. The Nosé-Hoover-Langevin piston method^25^ and Langevin dynamics were used for pressure control at 1 atm and temperature control at 300 K, respectively. Snapshots of the hSCGN and Ca^2+^-bound hSCGN trajectories were output every 10 ps and the 4000 structures from these trajectories were analyzed using VMD^26^ and Xmgrace^27^.

### Insulin secretion assay

Insulin secreted into the medium was measured using Mouse Ultrasensitive Insulin ELISA kit (ALPCO) following the manufacturer’s protocol. NIT-1 cells were starved at 37°C for 2 h with glucose-free HBSS (137 mM NaCl, 5.4 mM KCl, 1.26 mM CaCl_2_, 0.98 mM MgSO_4_, 0.44 mM KH_2_PO_4_, 0.36 mM Na_2_HPO_4_, 4.2 mM NaHCO_3_ (pH 7.4)) containing 0.1% BSA. After glucose starvation, the cells were stimulated at 37 °C for 25 min with HBSS containing 16.8 mM D-glucose. Secreted insulin was normalized to cellular total protein concentration, measured by a BCA protein assay performed according to the manufacturer’s instructions.

### Size exclusion chromatography

450 µg of protein was loaded on a Superdex^TM^ 200 HR 10/30 column (GE Healthcare) equilibrated with PBS. The column was eluted at a flow rate of 0.5 mL/min, and proteins were fractionated using FPLC (AKTA Explorer) into 15 fractions of 0.5 mL each. Proteins used as molecular weight markers were albumin (66.4 kDa) and α-lactalbumin (14.2 kDa).

### Immunoprecipitation from cell extracts

MDA-MB 231 cells were lysed with lysis buffer (150 mM NaCl, 50 mM Tris-Cl, 60 mM octyl β-D-glucopyranoside, pH 7.4) containing protease inhibitors for 30 min on ice. The cell lysates were then centrifuged at 12,000 rpm for 15 min at 4°C. The supernatant was incubated with anti-SCGN antibody for 2 h and then with protein G Sepharose beads for 1 h at 4°C. The beads were washed three times with 1 mL of lysis buffer and additionally twice with 1 mL of lysis buffer without detergent. The immune complex was solubilized in SDS gel sample buffer, separated on SDS-PAGE, and visualized by silver staining or Western analysis.

### Subcelluar fractionation

Cells (70–80% confluent in 100 mm dishes) were washed and collected by scraping into ice-cold PBS, pelleted by centrifugation at 4°C, resuspended in hypotonic buffer (10 mM Hepes, pH 7.2, 10 mM KCl, 1.5 mM MgCl_2_, and 100 μM Na_3_VO_4_), and a mixture of protease inhibitors (Roche) for 15 min on ice and disrupted using 32-gauge syringes. Crude nuclear fractions were pelleted by 5000 rpm for 10 min centrifugation at 4 °C, and supernatants were separated by 40,000 rpm for 1 h centrifugation at 4°C to cytosol (supernatants) and membrane (pellets).

## Results and Discussion

### Calcium ion binding promotes ROS-induced dimerization of hSCGN

**Figure 1:**
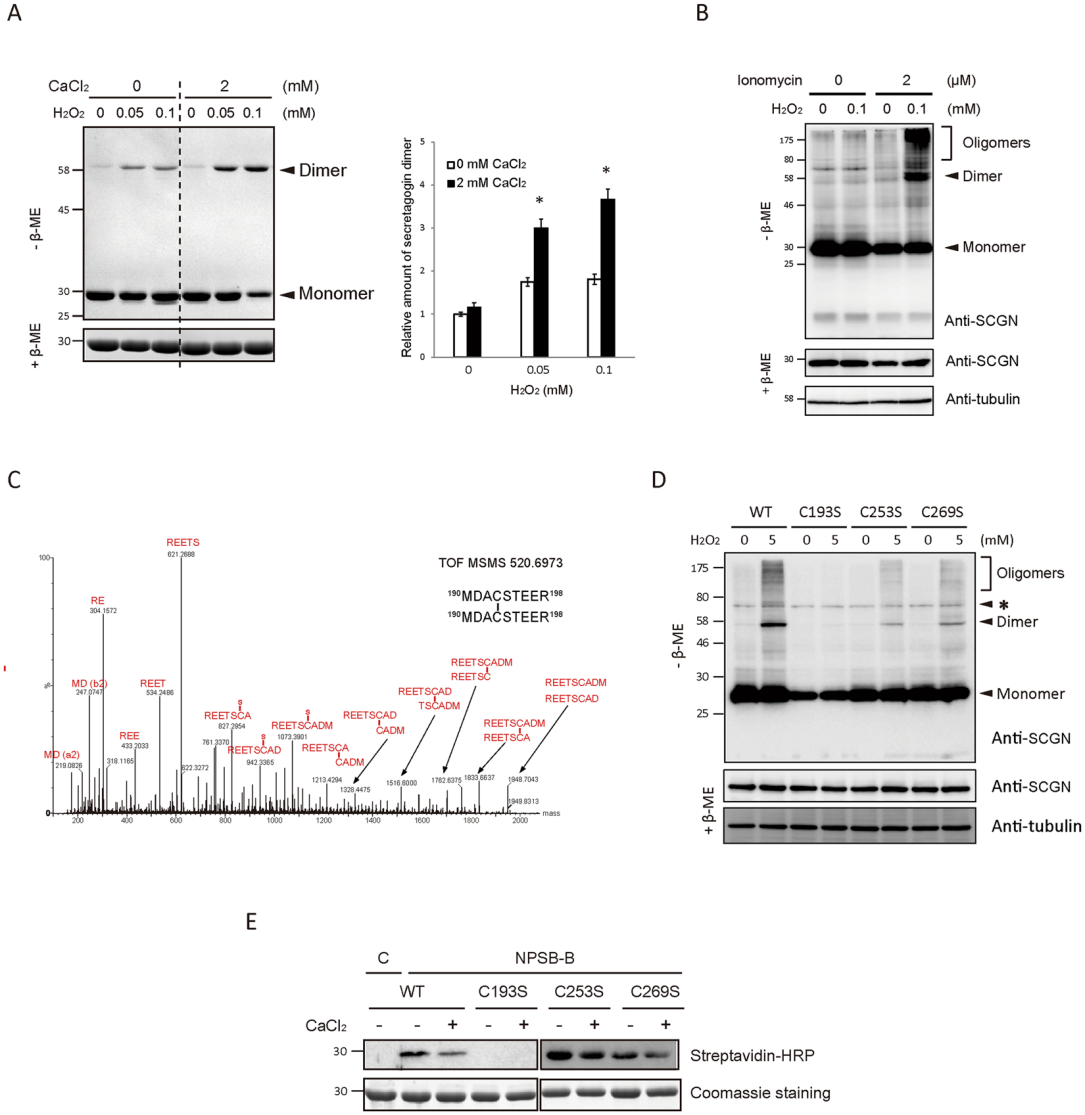
Calcium binding of hSCGN promotes H_2_O_2_ induced dimerization via C193-C193 disulfide linkage. (**A**) Recombinant hSCGN was incubated with 0 or 2 mM CaCl_2_ at R.T. for 15 min followed by H_2_O_2_ treatment in indicated concentration at 37°C for 1 h. Proteins were detected with coomassie blue-staining (left panel) and dimer formations were quantified and normalized with reduced form of SCGN (right panel). Data are presented as mean ± SD of three experiments (*P < 0.05, Student’s t-test). (**B**) NIT-1 insulinoma cells overexpressing hSCGN WT were pre-incubated with 0 or 2 μM ionomycin for 5 min followed by incubation with 0 or 100 μM H_2_O_2_ for 30 min at 37 °C. Total protein lysates were analyzed by Western blots using anti-SCGN or anti-α-tubulin antibody. (**C**) Tandem mass spectrum of C193-C193 disulfide linked peptide in band #7 and 8 of Supplementary Fig. S2A. (**D**) HeLa cells overexpressing SCGN WT, and Cys mutant (C193S, C253S, C269S) were treated with 0, 5 mM H_2_O_2_ for 10 min at 37°C. The total lysates were separated on non-reducing and reducing SDS PAGE and hSCGN and tubulin were detected by Western analysis. Tubulin was used for loading control. Non-specific bands were indicated as *. (**E**) hSCGN WT, and Cys mutants were incubated with 2 mM CaCl_2_ at R.T. for 15 min followed by treatment with 1 mM NPSB-B at R.T. for 2 h. Proteins were separated on reducing SDS-PAGE and detected by streptavidin-HRP. Coomassie staining gel showing amount of gel loaded proteins. Full-length gels and blots are in Supplementary Fig. S6.

In order to understand how hSCGN changes following Ca^2+^ binding, we first examined the electrophoretic mobility of recombinant hSCGN. Since SCGN is known to be partially oxidized during purification, by forming dimers through intermolecular disulfide bonds^28^, we investigated whether Ca^2+^ binding affects the dimerization of hSCGN. When recombinant hSCGN was incubated with CaCl_2_, small amounts of dimeric hSCGN were observed (Supplementary Fig. S1A), and the amount increased with incubation with H_2_O_2_ and Ca^2+^ (Fig. 1A). These findings indicate that Ca^2+^ and ROS synergize in the promotion of hSCGN dimerization. To further investigate the oligomerization state of hSCGN, the molecular mass of hSCGN after Ca^2+^ and H_2_O_2_ treatments were assessed by size exclusion chromatography (SEC). Samples were pretreated with EGTA and DTT to diminish dimerization due the possible effects of trace amounts of Ca^2+^ and air during the purification procedures. In the presence of Ca^2+^, H_2_O_2_ produced stable disulfide-linked dimers, while in the absence of Ca^2+^, less dimerization and more non-specific high molecular weight species were observed (Supplementary Fig. S1B). These results suggest that Ca^2+^ promotes the formation of a specific dimer by reducing non-specific high molecular weight oligomers, and that H_2_O_2_ is the driving force behind dimer formation.

These *in vitro* findings were validated in HeLa cells, which do not express endogenous hSCGN (Supplementary Fig. S1C), and confirmed by ProteinAtlas database search, (http://www.proteinatlas.org/ENSG00000079689-SCGN/cell), which showed SCGN mRNA was not detected in HeLa cells. When HeLa cells transfected with hSCGN were exposed to various concentrations of H_2_O_2_, the overexpressed hSCGN was found to dimerize in a dose- and time-dependent manner (Supplementary Fig. S1D). H_2_O_2_-induced hSCGN dimerization was increased by treatment of the cells with ionomycin, an agent that raises intracellular Ca^2+^ levels (Supplementary Fig. S1E). The effects of Ca^2+^ and oxidative stress on SCGN dimerization were also assessed in NIT-1 cells, a mouse insulinoma cell line expressing SCGN endogenously. We treated NIT-1 cells overexpressing SCGN with ionomycin, followed by H_2_O_2_ at previously determined optimal doses for NIT-1 cells^6^. SCGN was found to be dimerized and oligomerized by treatment with both ionomycin and H_2_O_2_ (Fig. 1B). These results show that Ca^2+^ binding enhances the H_2_O_2_-induced dimerization of hSCGN in both cell lines.

### Calcium ion-stimulated dimerization of hSCGN involves the formation of a Cys193-Cys193 inter-disulfide bond

Recombinant hSCGN was again pre-treated with DTT and EDTA to minimize background levels of dimerization due to traces of Ca^2+^ and the air environment. Supplementary Fig. S2A shows that uniform stable hSCGN dimerization was enhanced by H_2_O_2_ treatment of Ca^2+^-bound hSCGN, while several upper and lower bands representing non-specific crosslinking were detected in the absence of Ca^2+^. hSCGN possesses three highly conserved cysteine residues at Cys193, Cys253 and Cys269 (Supplementary Fig. S3A). We identified the disulfide linkages in hSCGN promoted by Ca^2+^ and H_2_O_2_ treatments, employing peptide sequencing with nanoUPLC-ESI-q-TOF mass spectrometry and the DBond disulfide searching algorithm, a unique algorithm for analysis of disulfide linkages directly in tandem mass spectra^28^. Disulfide linkages of each hSCGN band in Supplementary Fig. S2A were identified as noted above. The main dimer band (#7, 8) of hSCGN possessed a Cys193-Cys193 linkage. Non-specific lower or upper bands of both apo-hSCGN monomer and dimer (#9, 10, 11, and 12) were identified as Cys193-Cys269, and hSCGN monomer with Ca^2+^ as Cys253-Cys269 (Supplementary Fig. S2B). Tandem mass spectra of the identified hSCGN disulfide bonds are shown in Fig. 1C and Supplementary Fig. S2C. The identification of the dimer linkage in Ca^2+^-treated hSCGN as Cys193-Cys193 was confirmed employing three Cys mutants, C193S, C253S, and C269S (Supplementary Fig. S3B). The hSCGN C193S mutant could not form dimers, while the hSCGN C253S and C269S mutants could. Dimerization was increased by Ca^2+^ binding, indicating that Cys193 is driving the disulfide linkage in SCGN dimers, while Cys253 and Cys269 did not affect Ca^2+^-response of SCGN in recombinant proteins (Supplementary Fig. S3B), and in HeLa cells overexpressing hSCGN WT and mutants as well (Fig. 1D).

To understand how the dimerization of hSCGN is regulated by Ca^2+^, we measured the redox sensitivity of hSCGN in response to H_2_O_2_ employing NPSB-B, a novel specific biotin-labeling chemical probe, which reacts with ROS sensitive Cys-SH residues^21^. As shown in Supplementary Fig. S3C, apo-hSCGN has a highly reactive cysteine residue, which is readily labeled with NPSB-B. The labeling decreased after oxidation with low concentrations of H_2_O_2_ (as little as 0.01 mM), in a H_2_O_2_ dose-dependent manner. This indicates that the availability of reactive Cys-SH residues can be affected by low concentrations of H_2_O_2_. To elucidate which cysteine residue is reactive and whether it is regulated by Ca^2+^, we examined NPSB-B labeling of various cysteine mutants. The cysteine reactivity of hSCGN WT, C253S and C269S was reduced by Ca^2+^ treatment; in contrast, the C193S mutant could not be labeled with NPSB-B (Fig. 1E). This indicates that Cys193 is the redox sensitive Cys residue and the reactivity of Cys193 in WT, C253S and C269S mutants is reduced by Ca^2+^ binding. These results suggest the possibility that conformational changes induced by Ca^2+^ binding in hSCGN regulate the redox reactivity of Cys193 residue, and cause the formation of stable dimers, without non-specific promiscuous dimerizations of hSCGN that occur in response to H_2_O_2_.

### Calcium ion binding induces structural changes at N-terminal exposure to surface of hSCGN

**Figure 2:**
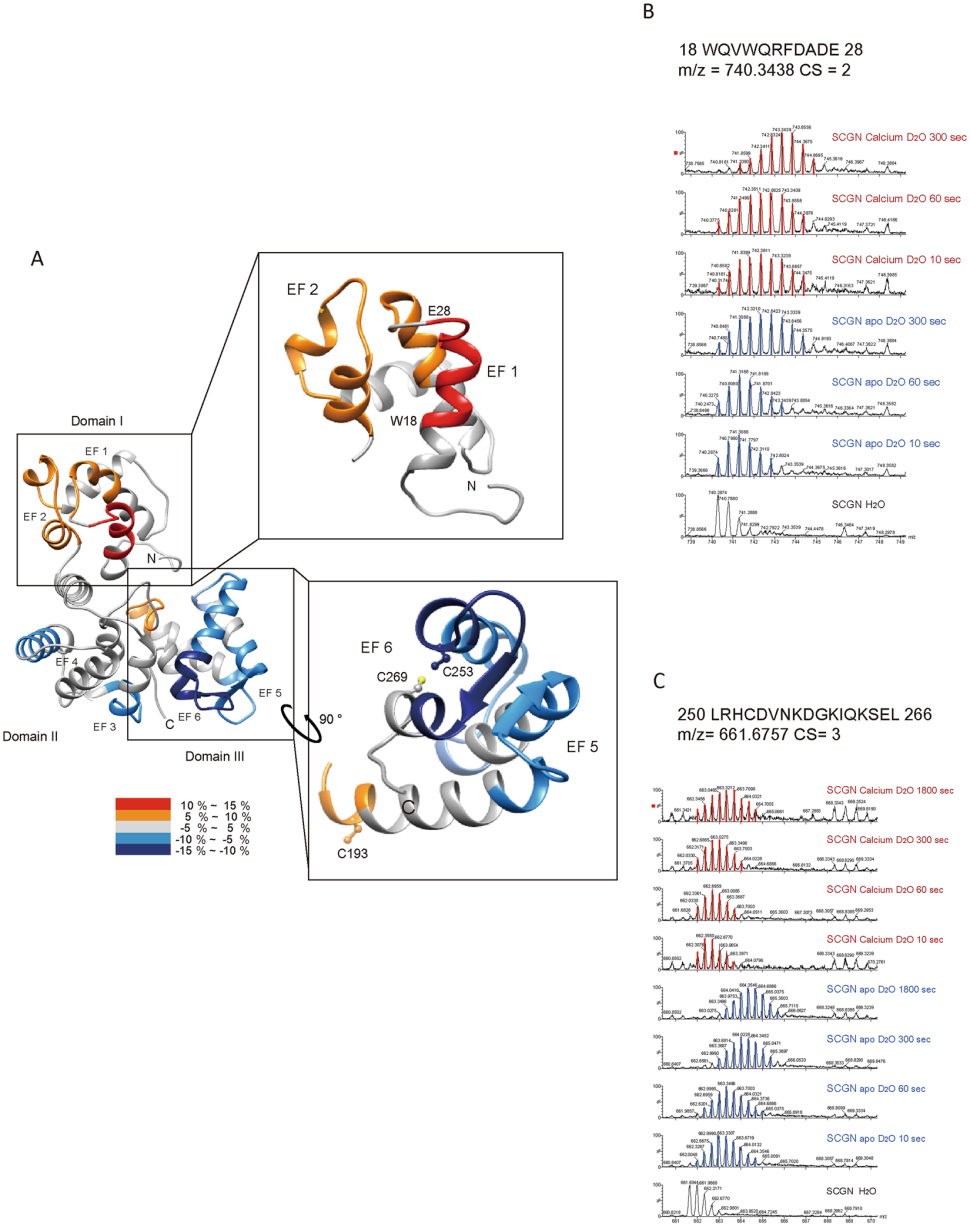
Structural changes in apo and Ca^2+^-bound hSCGN analyzed by hydrogen/deuterium exchange mass spectrometry. (**A**) Overlay of differential HDX data onto the structures of human SCGN structure which were modeled *in silico* with the MODELLER 9.9 program. Percentage difference in HDX between apo and Ca^2+^ bound hSCGN is colored according to the key. The differential deuterium exchange patterns of hSCGN peptide sequences representing EF-hand and Cys residue in N-terminal (**B**) and C-terminal (**C**) peptides of hSCGN.

Calcium sensor proteins having EF-hands are generally assumed to undergo conformational rearrangements following Ca^2+^ binding. These changes enable them to interact with target proteins and initiate signal transduction^14^. To determine whether structural changes occur in hSCGN in response to Ca^2+^ binding and thus potentially regulate hSCGN function, we conducted HDX-MS analysis of apo and Ca^2+^-bound SCGN. Because the crystal structure of calcium-bound hSCGN could not be obtained due to failure of crystal formation, we mapped hSCGN sequence onto the structure deduced from x-ray crystallographic and biophysical studies of *Danio rerio* SCGN in its Ca^2+^-free apo-state^19^, and used this structure to display the results of the HDX-MS studies. These studies revealed clear differences between apo and Ca^2+^-bound hSCGN with respect to the deuterium incorporation rates in particular regions of the protein (Supplementary Table S1). There were significant conformational changes in domains I and III of hSCGN (Fig. 2A). In Domain III, Ca^2+^ binding reduced the deuterium incorporation of EF5 and EF6 (Fig. 2C), and increased it at the Cys193 residue. The most dramatic HDX changes were found in N-terminal residues in EF1 and EF2 (18–28 a.a.) of Domain I; upon Ca^2+^ binding, there was a substantial increase in deuterium uptake of these hydrophobic residues (Fig. 2B). This indicates that Ca^2+^ binding causes significant conformational changes within the N-terminal region which could impact Cys193 and increase the dimerization of hSCGN. Ca^2+^ induced exposure of a hydrophobic patch seems to be a common feature of Ca^2+^ binding proteins. The structures of domains II and III of SCGN are superimposable upon the corresponding EF-hands of calbindin D28K, while that of domain I is not^19^. SCGN is known to have three isoforms, two of which are characterized by single amino acid changes at residue 22 Gln/Arg, and setagin is a variant of SCGN that consists of 49 amino acids having identical N-terminal 27 residues^29^. The N-terminal region of SCGN is presumed to have a distinct role in cellular processes, based on its structure and isoforms. Regulatory EF-hand proteins are believed to undergo conformational changes upon Ca^2+^ binding, which affect various cellular functions. However, the details of the underlying mechanisms are not well understood. This study clearly shows that SCGN undergoes large conformational changes triggered by Ca^2+^ binding, which may play a role in Ca^2+^ dependent dimerization of hSCGN.

**Figure 3:**
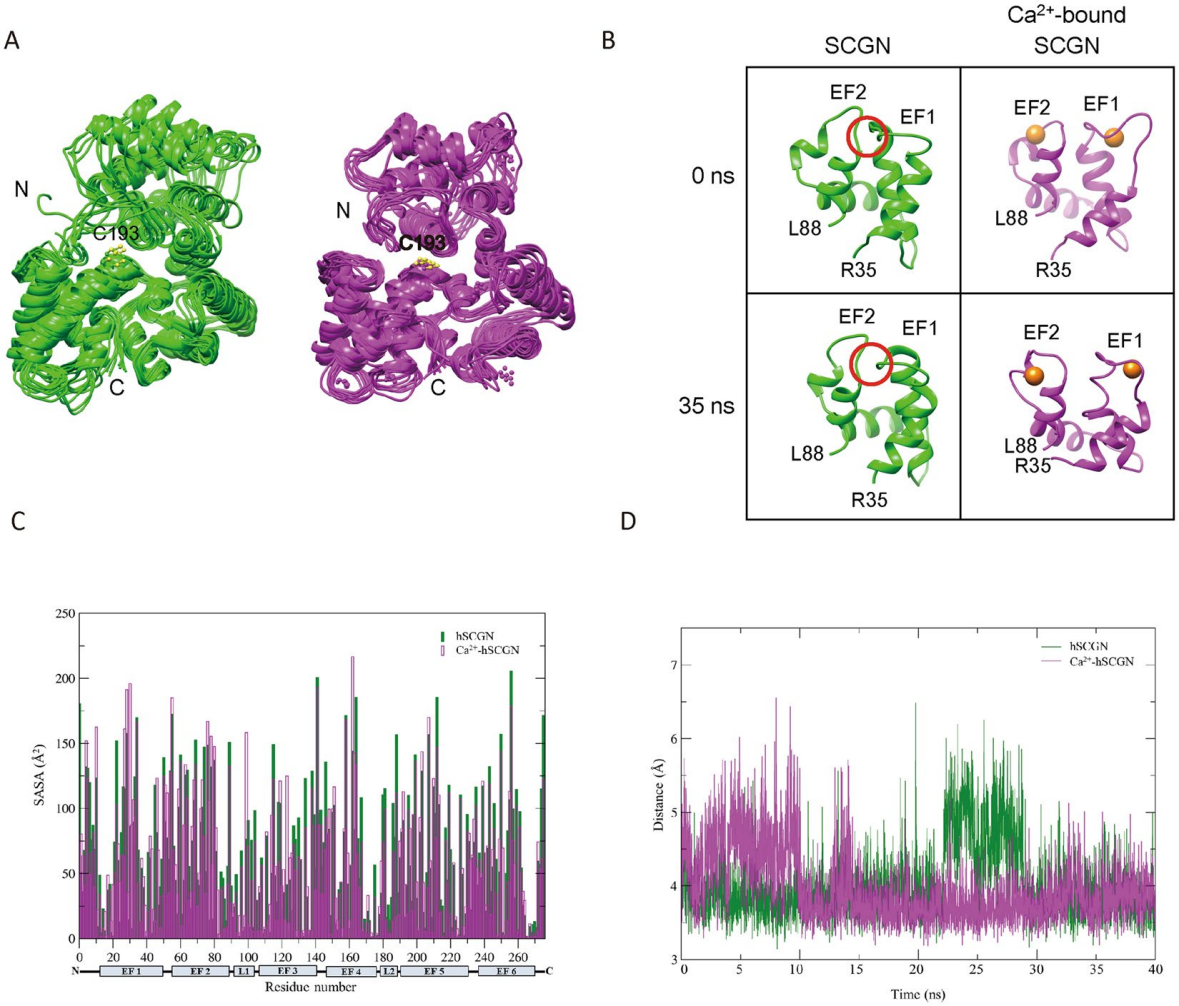
Dynamics of apo and six Ca^2+^-bound hSCGN in molecular dynamics simulation. (**A**) The coordinates of apo-hSCGN and Ca^2+^-bound hSCGN saved every 5 ns during MD simulations are superimposed, respectively. (**B**) Snapshots of the domain I of apo-hSCGN and Ca^2+^-bound hSCGN at 0 ns and 35 ns. Ca^2+^ ions are drawn as orange spheres. The conformation of the red circled area is a β-sheet at 0 ns when analyzed by STRIDE (http://webclu.bio.wzw.tum.de/cgi-bin/stride/stridecgi.py). (**C**) The average SASA values for every residue of 400 structures saved every 100 ps during 40 ns simulation. (**D**) The distances between Cys253 and Cys269 during simulation for apo-hSCGN (green) and Ca^2+^-bound hSCGN (magenta).

To further corroborate these HDX-MS results, we explored the dynamics of hSCGN and the events occurring in the Ca^2+^ binding, using molecular dynamics simulations. The starting hSCGN structure was generated using the MODELLER 9.9 program with the 3D structure of SCGN from *Danio Rerio* ^19^ as a template. We performed equilibrium molecular dynamics (MD) simulations of hSCGN with and without six Ca^2+^ ions (Fig. 3A). The six calciums were modeled in accordance with the sequence preference of the EF-hand loop (Supplementary Table S2)^23^. Root mean standard deviations (RMSDs) were calculated with respect to the backbone coordinates of the starting structures, with and without six Ca^2+^ ions (Supplementary Fig. S4A), respectively. Both structures changed significantly during the first 5 ns. However, the changes in the Ca^2+^-bound form reached the equilibrated trajectories more quickly, corresponding to an RMSD of slightly higher than 3 Å. In contrast, RMSDs of the Ca^2+^-free form surged up to ~4 Å and then decreased to ~2.8 Å in 25 ns. The sudden increase of RMSDs at 40 ns originated from flexibility of ten N-terminal residues in the absence of Ca^2+^ together with motion of Domain I, consistent with the results of the HDX studies. In the presence of 2 mM Ca^2+^, the interface between EF1 and EF2 was exposed, again as shown in the HDX experiments. MD simulations also showed that the conformational change at the interface was negligible in the absence of Ca^2+^, but was significant in the presence of Ca^2+^ (Fig. 3B). The binding of Ca^2+^ to EF1 and EF2 affected the short β-strands in the latter part of each loop, which form a small β-sheet. As a result, the interface gradually became more solvent-accessible, as shown in the solvent accessible surface area (SASA) plot (Fig. 3C). MD simulations also showed that the distance between two sulfur atoms of Cys253 and Cys269 became equilibrated after 15 ns with an average distance of ~3.7 Å (Fig. 3D) in the Ca^2+^-bound form. Since Cys253 and Cys269 are present on EF6, the binding of Ca^2+^ in EF6 appears to stabilize the region around these residues. Together with the increasing positive potential generated by Ca^2+^, the environment seems appropriate to form a disulfide bond between these two residues. However, the average distance between the sulfur atoms of Cys253 and Cys269 in the Ca^2+^-free form showed intermittent fluctuations even at the equilibrated state due to dynamic motion of EF6. This could make the formation of the disulfide bond between Cys253 and Cys269 difficult in the Ca^2+^-free form, in agreement with the results from MS analysis. Based on disulfide analysis by MS (Supplementary Fig. S2), the tendency for Cys253-Cys269 intra-disulfide bond formation in the monomer was enhanced by Ca^2+^ binding at band 2 and 6, and NPSB-B labeling and stable dimer formation of Cys253 or Cys269 mutants was increased (Fig. 1D). Thus the Cys253-Cys269 intramolecular disulfide bond is not essential for dimerization of SCGN, although it might facilitate the stable dimerization of hSCGN by solidifying C-terminal structure via Cys253-Cys269 linkage.

Rogstam *et al*.^13^ reported four calcium binding sites, one with high affinity and three with similar affinities. The explanations for our adding two more Ca^2+^ ions in the putative EF1 and EF2 domains were based on our studies of HDX with 2 mM CaCl_2_ where we found a vivid surface exposure with EF1 and EF2. It is evident that the two N-terminal EF1 and EF2 have lower calcium affinity compared with other four EFs. However, EF1 and EF2 are predicted as an EF-motif in EF-motif prediction programs such as CAL-EF-AFi (http://202.41.10.46/calb/index.html) and conformational changes at 2 mM CaCl_2_ have been clearly shown in the HDX experiments. Our experiments showed that EF1 and EF2 bind to Ca^2+^ at high calcium concentration due to their low calcium affinity. And our studies on MD simulation with six instead of four Ca^2+^ ions are in agreement with the above. Since there is no other calcium ion binding site in the N-terminal domain, the only plausible explanation of the conformational change around EF1 and EF2 was the binding of calcium ions in these EF motifs. Therefore, HDX experiment proves that hSCGN binds 6 Ca^2+^ in high Ca^2+^ concentration.

A dimeric hSCGN was constructed from coordinates at 35 ns, using HEX 6.3^30^. The Ca^2+^-bound dimeric form clearly showed that the dimeric interface is well formed and that the N-terminal domain is located on the same side as Cys193 (Supplementary Fig. S4B). In addition, the two Cys193 residues face each other, corresponding with the Cys193-Cys193 dimer identified by MS. However, formation of a dimeric interface was not feasible with the Ca^2+^-free form taken from the 35 ns coordinates; and its N-terminal loop, as it clashed with the other subunit, if superimposed on the dimeric form of the Ca^2+^-bound form. Therefore, a decrease in the dynamic motion of the N-terminal domain may be an important factor for dimer formation. These results support the suggestion that the dimeric hSCGN is increased in the presence of Ca^2+^.

### N-terminal region regulates Cys193-Cys193 dimer formation of hSCGN

**Figure 4:**
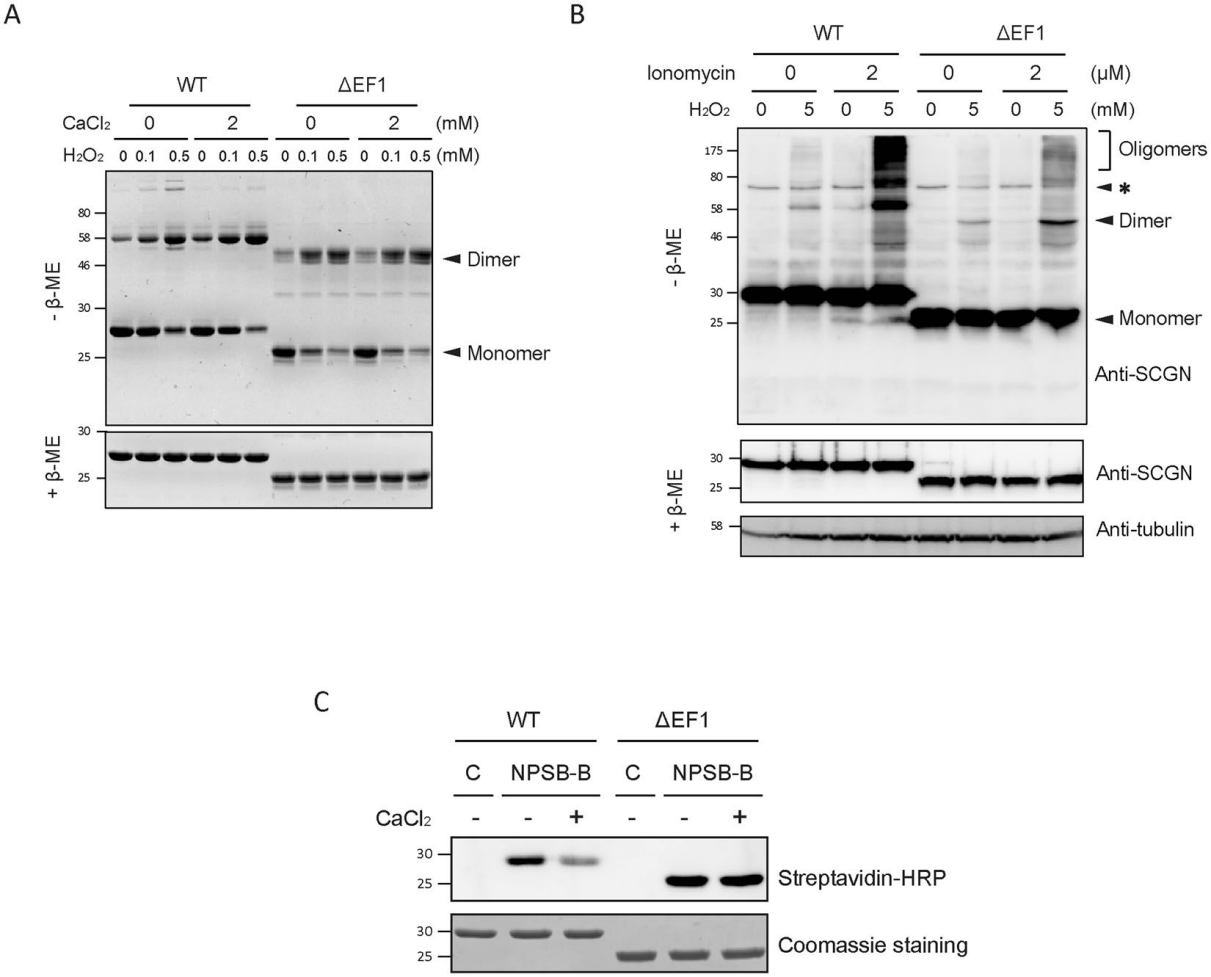
N-terminus of hSCGN is essential for calcium response. (**A**) Purified hSCGN WT and ΔEF1 protein were pre-treated with 0, 2 mM CaCl_2_ for 15 min at R.T. and then incubated with the indicated concentrations of H_2_O_2_ for 1 h at 37°C. Coomassie blue-stained SDS-PAGE gels under non-reducing (−β-ME) and reducing (+β-ME) condition. (**B**) HeLa cells overexpressing hSCGN WT and ΔEF1 were treated with 0 or 2 μM ionomycin for 5 min and with 0 or 5 mM H_2_O_2_ for 30 min at 37 °C. Cell lysates were subjected to Western analysis using anti-hSCGN or anti-α-tubulin antibody. Non-specific bands were indicated as *. (**C**) Recombinant hSCGN WT and ΔEF1 proteins were incubated with 0 or 2 mM CaCl_2_ for 15 min, and with 1 mM NPSB-B for 2 h at R.T. Labeled samples in gel sample buffer containing 10 mM NEM were detected with streptavidin-HRP. Full-length gels and blots are in Supplementary Fig. S7.

Since Ca^2+^ binding induces structural changes in the N-terminal region of hSCGN, we hypothesized that N-terminal exposure regulates the intermolecular dimerization of Cys193 in Ca^2+^-bound hSCGN. To test this hypothesis, we generated a deletion mutant of hSCGN (ΔEF1, deletion of 1–52 a.a.), which has the stable conformation of purified protein. We examined the effect of truncating EF1 on Ca^2+^ triggered dimer formation. As shown in Fig. 4A, dimerization of ΔEF1 was not increased by Ca^2+^, while H_2_O_2_ induced dimerization was not affected by EF1 deletion. This is consistent with the hypothesis that a conformation change in the N-terminal region mediates the Ca^2+^-induced dimerization of hSCGN. To further test these results in *in vivo*, we examined hSCGN dimer formation in HeLa cells. HeLa cells were transfected with hSCGN WT or ΔEF1 and were pre-incubated with ionomycin for 5 min to raise the intracellular Ca^2+^ concentration, followed by oxidative stimulation with H_2_O_2_ for 30 min. Dimer formation in each case was determined by Western analysis under non-reducing conditions. As shown in Fig. 4B, the amount of dimerization in response to ionomycin and H_2_O_2_ treatments was significantly higher in cells overexpressing hSCGN WT than the ΔEF1 mutant.

To further test whether N-terminal structural changes caused by Ca^2+^ binding (Fig. 2) affect the reactivity of Cys193, we examined the reactivity of Cys193 in transfected WT and ΔEF1 hSCGN using the chemical probe NPSB-B. NPSB-B labeling of hSCGN WT was significantly decreased upon ionomycin treatment, while that of ΔEF1 mutant did not change (Fig. 4C). This indicates that the ΔEF1 mutant, in contrast to hSCGN WT, cannot regulate the reactivity of Cys193 of hSCGN in response to Ca^2+^. These results are again consistent with the hypothesis that the conformational changes in the N-terminal domain of hSCGN caused by Ca^2+^ binding play a critical role in the regulation of dimerization and Cys193 reactivity.

### Cys193-dependent dimerization of SCGN is an essential step regulating insulin secretion in pancreatic beta cells

**Figure 5:**
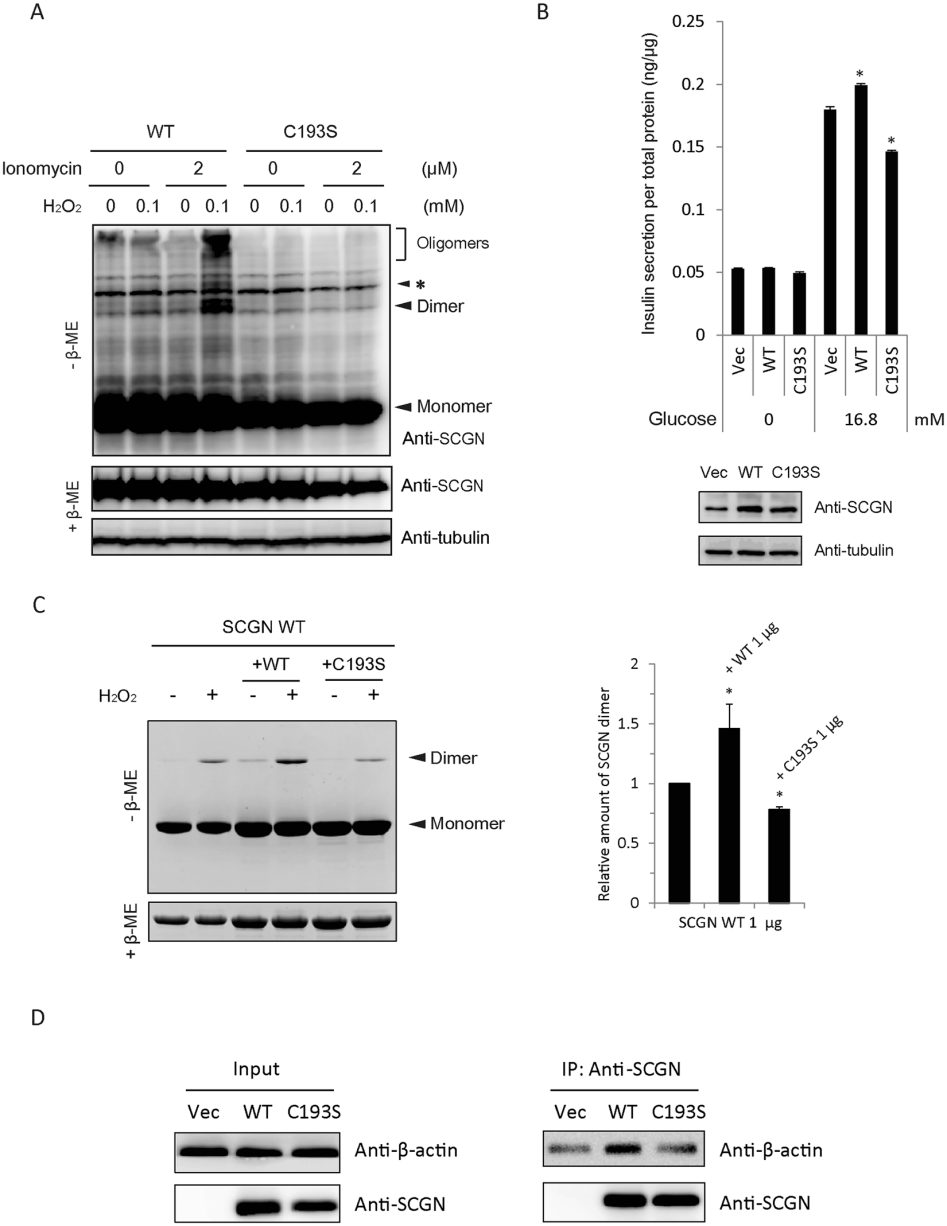
Dimerization of hSCGN is required for its role in insulin secretion. (**A**) NIT-1 insulinoma cells overexpressing hSCGN WT and C193S mutant were pre-incubated with 0 or 2 μM ionomycin for 5 min followed by incubation with 0 or 100 μM H_2_O_2_ for 30 min at 37 °C. Proteins were separated under non-reducing and reducing conditions on SDS PAGE and detected with Western analysis using anti-SCGN antibody. Non-specific bands were indicated as *. (**B**) NIT-1 cells overexpressing hSCGN WT or C193S mutant were starved with glucose-free HBSS for 2 h followed by stimulation with 16.8 mM glucose for 25 min. The amount of secreted insulin was measured and normalized to total cell protein concentration. Whole cell lysates were subjected to Western analysis with anti-SCGN or anti-α-tubulin antibody. (**C**) Dimer formations of hSCGN WT, and hSCGN WT mixed with same amount of hSCGN WT or C193S proteins were measured after treating with 2 mM CaCl_2_ for 15 min at R.T. followed by incubation with 0 or 1 mM H_2_O_2_ for 1 h at 37°C. Proteins were detected with coomassie blue-staining (left panel) and were quantified and normalized with reduced form of SCGN (right panel). (**D**) hSCGN C193S fails to interact with cytoskeletal actin in MDA-MB-231 cells. MDA-MB-231 cells overexpressing SCGN WT and C193S mutant were lysed with a lysis buffer containing protease inhibitors. Cell lysates were immunoprecipitated with anti-SCGN antibody and the immune complexes were separated on SDS-PAGE and detected with western analysis using anti-actin and anti-SCGN antibodies. Data information: In (**B**,**C**), data are expressed as the mean ± SD of three experiments (*P < 0.05, Student’s t-test). Full-length gels and blots are presented in Supplementary Fig. S8.

To determine the interrelationships among Ca^2+^ binding, structural changes, dimerization of SCGN, and glucose-stimulated insulin secretion (GSIS), we examined insulin secretion in NIT-1 cells, a mouse insulinoma cell line that expresses SCGN endogenously^31–33^. We first assessed the effect of Ca^2+^ and oxidative stress on SCGN dimerization in NIT-1 cells. We treated NIT-1 cells transfected with SCGN WT and C193 mutant with ionomycin, followed by H_2_O_2_ at doses that have been previously determined to be the physiologically relevant and optimal for NIT-1 cells^6^. WT hSCGN was found to be dimerized and oligomerized by treatment with ionomycin plus H_2_O_2_, while C193S hSCGN did not (Fig. 5A). No increase in dimerization of SCGN was found on treatment with H_2_O_2_ or ionomycin alone, suggesting that Ca^2+^ binding is necessary for the formation of dimers and oligomers and indicating that there is a strong synergy between the two treatments.

Since glucose stimulation promotes intracellular Ca^2+^ influx and ROS generation, we investigated whether SCGN dimerization contributes to glucose stimulated insulin secretion (GSIS) in pancreatic β-cells. NIT-1 cells transiently transfected with control vector, hSCGN WT and C193S mutant were starved for 2 h, followed by stimulation with glucose for 30 min and assay of secreted insulin. Figure 5B shows that GSIS was augmented in hSCGN WT transfected cells compared to control. hSCGN WT has a positive effect on insulin secretion, while the C193S mutant, unable to form dimers, partially inhibited it. To further explain this inhibitory effect of the C193S mutant on insulin secretion, we examined the effect of the C193S mutant on the dimerization of hSCGN WT using recombinant proteins. As shown in Fig. 5C, dimerization of hSCGN was reduced by addition of the C193S mutant, indicating that C193S interrupts hSCGN WT dimer formation. These results suggest that Cys193-dependent dimerization of SCGN is an essential step for regulating insulin secretion and that C193S mutant may act as a dominant negative mutant by disturbing the dimerization of endogenous SCGN in NIT-1 cells. Although the overexpression of hSCGN was modest because of the high endogenous SCGN expression in NIT-1 cells, the differences in insulin secretion between cells overexpressing hSCGN WT and C193S mutants vs. the controls were statistically significant.

We previously reported that hSCGN regulates insulin secretion through interaction with the actin cytoskeleton^6^. hSCGN interacts with actin and this interaction was increased by H_2_O_2_ and glucose stimulation, indicating that Ca^2+^ and ROS signaling regulate the interaction between SCGN and actin. To determine whether C193S hSCGN interferes with actin binding, we performed immunoprecipitation with hSCGN WT and C193S mutant. Since NIT-1 cells are not suitable for immunoprecipitation with ectopic hSCGN because of their high endogenous expression of SCGN, we used MDA-MB-231 cells, which express only trace amounts of hSCGN. We found that the C193S mutant did not interact with the actin cytoskeleton, whereas hSCGN WT did (Fig. 5D). These results suggest that SCGN dimerization plays a role in regulating insulin secretion through interaction with actin cytoskeleton.

We previously reported that SCGN regulates GSIS via ROS signaling and H_2_O_2_ treatment enhanced the co-localization with actin cytoskeleton in plasma membrane^6^. The dimerization of SCGN has a modulatory role in insulin secretion and further oligomerization of SCGN occurs only inside cells in response to H_2_O_2_ and ionomycin (Figs 1B,D, 4B and 5A). We examined whether dimerization and oligomerization are crucial for localization to membrane fraction, we performed fractionation of ectopic WT and Cys mutants of SCGN in HeLa cells. Supplementary Fig. S5 shows SCGN monomer and dimer localized to cytosol, nuclear, and membrane fractions, while SCGN oligomer localized in only nuclear and membrane fractions. C193S failed to form the dimer, therefore, dimeric and oligomeric C193S SCGN did not localized to nuclear and membrane fractions, indicating that dimerization is prerequisite for oligomerization. It is possible that SCGN dimers form hetero-oligomers with vesicle secretory machinery in NIT-1 cells in response to glucose stimulation, or that other special cellular conditions are required for oligomerization^34^. It is also possible that glucose stimulation increases the intracellular Ca^2+^ concentration and acutely dimerizes Ca^2+^-bound SCGN by ROS generation, with the dimerized SCGN forming oligomers that bind with the actin cytoskeleton to enhance insulin secretion. However, cellular function of oligomers remains to be further elucidated.

In summary, this study identifies a novel regulatory mechanism in the regulation of insulin secretion in pancreatic β-cells by SCGN that involves stepwise structural changes brought on by Ca^2+^ binding, followed by SCGN dimerization by cysteine oxidation in response to ROS. Moreover, these changes appear to be important for GSIS. The elevation of intracellular Ca^2+^ concentration in glucose-stimulated pancreatic β-cells is hypothesized to cause conformational changes in inactive apo-SCGN via Ca^2+^ binding by inducing N-terminal flexibility. This is followed by allosteric regulation of Cys193 reactivity and stabilization of C-terminal disulfide bonds between C253 and C269 that prevents non-specific dimerization and induces an N-terminal conformation change to form Cys193-linked dimers more efficiently. This study identifies the molecular crosstalk between Ca^2+^ binding and oxidation in the formation of functionally-important disulfide dimers, crucial for the biological function of SCGN in insulin secretion in pancreatic β-cells.

## Electronic supplementary material

Supplementary Information
