# Supplementary material for: Calcium Ion Induced Structural Changes Promote Dimerization of Secretagogin, Which Is Required for Its Insulin Secretory Function
Source: Sci Rep. 2017 Aug 1;7:6976. doi: 10.1038/s41598-017-07072-4 (PMC5539292; doi:10.1038/s41598-017-07072-4)
Supplement: Supplementary file 1 — Supplementary Information [file 41598_2017_7072_MOESM1_ESM.pdf]

Supplementary Information

Calcium Ion Induced Structural Changes Promote Dimerization of Secretagogin, Which Is Required for Its Insulin Secretory Function

Jae-Jin Lee[1]*, Seo-Yun Yang[1]*, Jimin Park[1], James E. Ferrell, Jr[2], Dong-Hae Shin[1] and Kong-Joo Lee[1†]

[1]Graduate School of Pharmaceutical Sciences, College of Pharmacy, Ewha Womans University, Seoul, Korea 120-750

[2]Department of Chemical and Systems Biology, Stanford University School of Medicine, Stanford, CA 94305-5174, USA

[*]These authors contributed equally to this work

†Address for correspondence: Kong-Joo Lee, Tel: +82-2-3277-3038, Fax: +82-2-3277-3760, e-mail: kjl@ewha.ac.kr

Supplementary Figure legends

Supplementary Figure 1 – SCGN is readily dimerized with disulfide linkage following calcium binding.

(A) Recombinant SCGN was incubated with indicated $CaCl_2$ concentration at R.T. for 15 min followed by 0.1 mM H_2O_2 treatment at 37℃ for 1 h. Data are presented as mean ± SD of three experiments (**P* < 0.05, Student's t-test). (B) Size exclusion chromatograms of SCGN protein in response to calcium and H_2O_2 treatments. SCGN protein treated with 0, 2 mM $CaCl_2$ for 15 min at room temperature followed by incubation with 0, 1 mM H_2O_2 for 1 h at 37℃. Apo- SCGN without H_2O_2 (black dotted line), Ca^{2+}-bound SCGN (2 mM $CaCl_2$) without H_2O_2 (red dotted line), Apo- SCGN treated with 1 mM H_2O_2 (black line), Ca^{2+}-bound SCGN with 1 mM H_2O_2 (red line). Albumin (66.4 kDa) and α-lactalbumin (14.2 kDa) were used as molecular weight markers. (C) Endogenous SCGN levels were detected in lung fibroblast WI38, cervical cancer cell line HeLa, lung cancer cell line, H358, H157, human embryonic kidney cell line HEK 293T, and mouse insulinoma cell line, NIT-1. The cell lysates were separated on reducing and reducing SDS-PAGE and SCGN was detected by WB. GAPDH was detected for loading control. Non-specific bands were indicated as * (D) SCGN forms dimers and oligomers in HeLa cells in response to H_2O_2. HeLa cells overexpressing SCGN were treated with the indicated H_2O_2 concentration for indicated times. The cell lysates were separated on non-reducing and reducing SDS-PAGE and SCGN was detected by WB. Tubulin was detected for loading control. (E) HeLa cells overexpressing SCGN were treated with the indicated H_2O_2 and ionomycin concentration for 30 min. Proteins were separated under non-reducing and reducing conditions on SDS-PAGE and SCGN n and tubulin were detected with western analysis.

Supplementary Figure 2 - Calcium bound SCGN forms stable dimers through C193-C193 disulfide linkage.

(A) Recombinant SCGN was incubated with 0 or 2 mM $CaCl_2$ at R.T. for 15 min followed by 1 mM H_2O_2 treatment for 1 h. Proteins were separated by non-reducing SDS-PAGE, and each protein bands were analyzed by peptide sequencing with nanoUPLC-ESI-Q-TOF using the DBond disulfide searching algorithm. (B) List of identified disulfide linkages of each SCGN band. (C) Tandem mass spectra of C193-C269, and C253-C269 disulfide linked peptide.

Supplementary Figure 3 - SCGN forms stable dimers via C193-C193 disulfide linkage and has a reactive cysteine readily oxidized by H_2O_2.

(A) Conservation map of cysteine residues in SCGN was obtained from the orthologs present in all of the animal species for which KEGG has complete genomes. Orthologies were manually checked if it is ambiguous. (B) SCGN WT, and Cys mutant (C193S, C253S, C269S) proteins were incubated with/without $CaCl_2$ at R.T. for 15 min followed by H_2O_2 treatment in indicated concentration at 37°C for 1 h. Proteins were separated by non-reducing and reducing SDS-PAGE. (C) Recombinant SCGN was incubated in indicated concentration at 37°C for 1 h. Proteins were incubated with 1 mM NPSB-B at R.T. for 2 h for labeling the reactive Cys residue, followed by separation on reducing SDS-PAGE and detection by streptavidin-HRP. Coomassie staining gel showing amount of gel loaded proteins.

Supplementary Figure 4 - Results of MD simulations.

(A) The RMSDs of backbone Cα atoms during the simulations for apo- SCGN (green) and Ca^{2+}-bound SCGN (magenta). (B) The dimeric Ca^{2+}-bound SCGN constructed with coordinates at 35 ns using HEX 6.3.

Supplementary Figure 5- Celluar localization of SCGN monomer, dimer, and oligomers

Subcelluar fractionation of Hela cells overexpressing SCGN WT, and Cys mutants (C193S, C253S, C269S) was performed and each fraction was separated on non-reducing and reducing SDS PAGE

and hSCGN was detected by Western analysis. PRDX6, lamin B, and flotillin, were used as markers for cytosol and nucleus, and membrane respectively.

Supplementary Figure 6 - Full size gels used in Figure 1

Supplementary Figure 7 - Full size gels used in Figure 4

Supplementary Figure 8 - Full size gels used in Figure 5

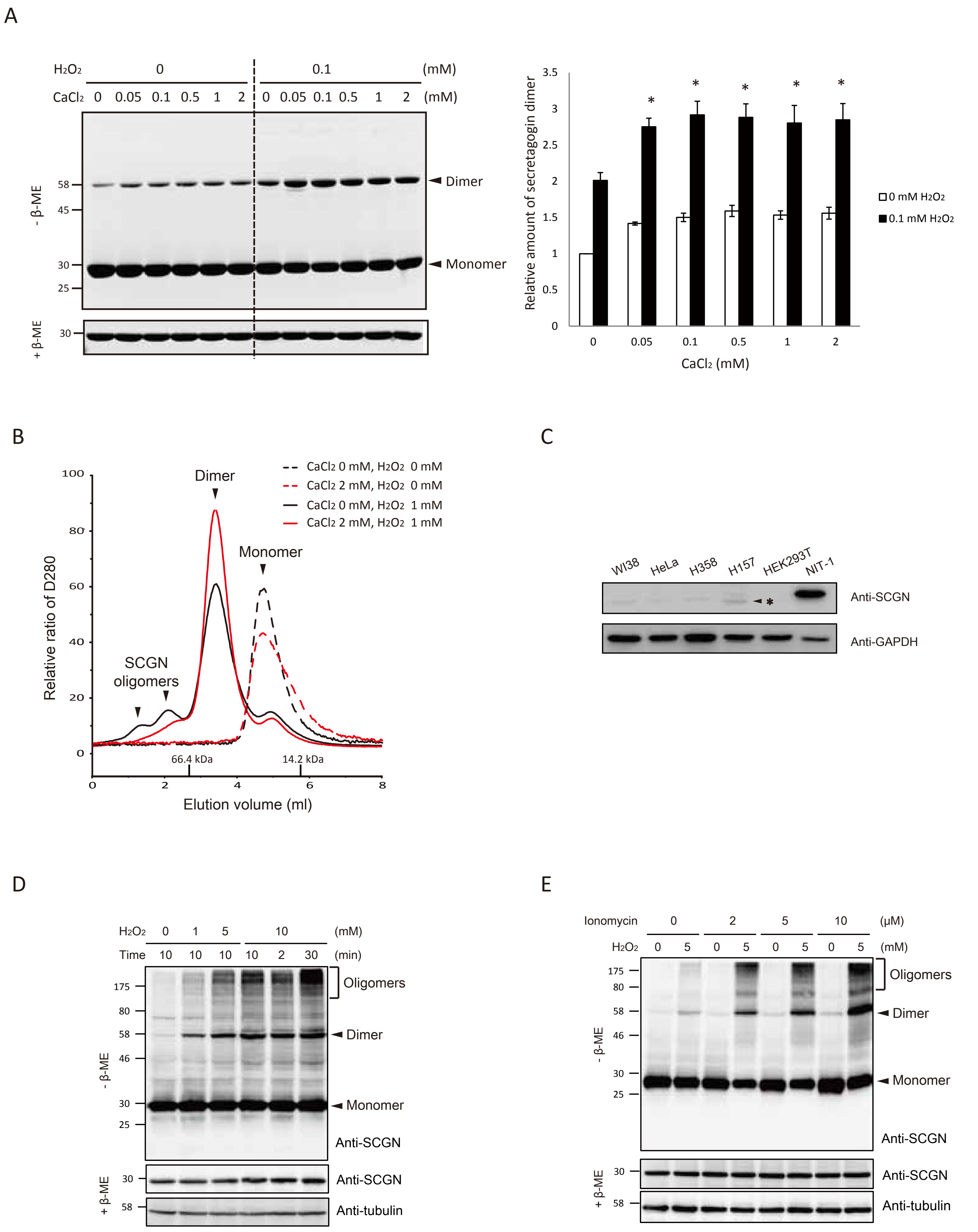

A
H2O2 0 0.1 (mM)
CaCl2 0 0.05 0.1 0.5 1 2 0 0.05 0.1 0.5 1 2 (mM)
- β-ME
58
45
30
25
Dimer
Monomer
+ β-ME
30
Relative amount of secretagogin dimer
3.5
3
2.5
2
1.5
1
0.5
0
0 0.05 0.1 0.5 1 2
CaCl2 (mM)
0 mM H2O2
0.1 mM H2O2
B
Dimer
Monomer
SCGN oligomers
CaCl2 0 mM, H2O2 0 mM
CaCl2 2 mM, H2O2 0 mM
CaCl2 0 mM, H2O2 1 mM
CaCl2 2 mM, H2O2 1 mM
Relative ratio of D280
100
80
60
40
20
0
66.4 kDa
14.2 kDa
0 2 4 6 8
Elution volume (ml)
C
WI38 HeLa H358 H157 HEK293T NIT-1
Anti-SCGN
Anti-GAPDH
D
H2O2 0 1 5 10 (mM)
Time 10 10 10 10 2 30 (min)
175
80
58
46
30
25
- β-ME
Oligomers
Dimer
Monomer
Anti-SCGN
+ β-ME
30
58
Anti-SCGN
Anti-tubulin
E
Ionomycin 0 2 5 10 (μM)
H2O2 0 5 0 5 0 5 0 5 (mM)
175
80
58
46
30
25
- β-ME
Oligomers
Dimer
Monomer
Anti-SCGN
+ β-ME
30
58
Anti-SCGN
Anti-tubulin

Figure S1

A

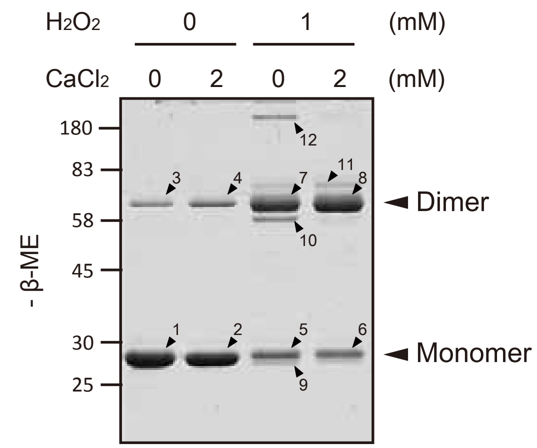

B

List of identified disulfide linkages of each SCGN band

Band No.	Description	H_2O_2	Ca^{2+}	Disulfide linkage	Observed mw (CS)	Calculated mw	DeltaM	Dbond Score
1	Monomer	-	-	No ID				
2	Monomer	-	+	Cys253 - Cys269	586.9968(3+)	1757.8804	0.0882	33.9
3	Dimer	-	-	Cys253 - Cys269	586.9984(3+)	1757.8804	0.093	7.3
4	Dimer	-	+	Cys253 - Cys269	586.9981(3+)	1757.8804	0.0921	2.8
5	Monomer	+	-	No ID				
6	Monomer	+	+	Cys253 - Cys269	586.9966(3+)	1757.8804	0.0876	36.4
7	Dimer	+	-	Cys193 - Cys193	520.6976(4+)	2078.7649	-0.0036	159.3
8	Dimer	+	+	Cys193 - Cys193	520.6973(4+)	2078.7649	-0.0048	223
9	Lower shifted Monomer	+	-	Cys193 - Cys269	525.9949(4+)	2099.9537	-0.0032	17.2
10	Lower shifted Dimer	+	-	Cys193 - Cys269	525.9955(4+)	2099.9537	-8.0E-4	45.6
11	Upper shifted Dimer	+	+	Cys193 - Cys269	700.9935(3+)	2099.9537	0.005	7
				Cys253 - Cys269	586.9656(3+)	1757.8804	-0.0054	9.2
12	Upper shifted Dimer	-	+	Cys193 - Cys269	700.9938(3+)	2099.9537	0.0059	18.9

C

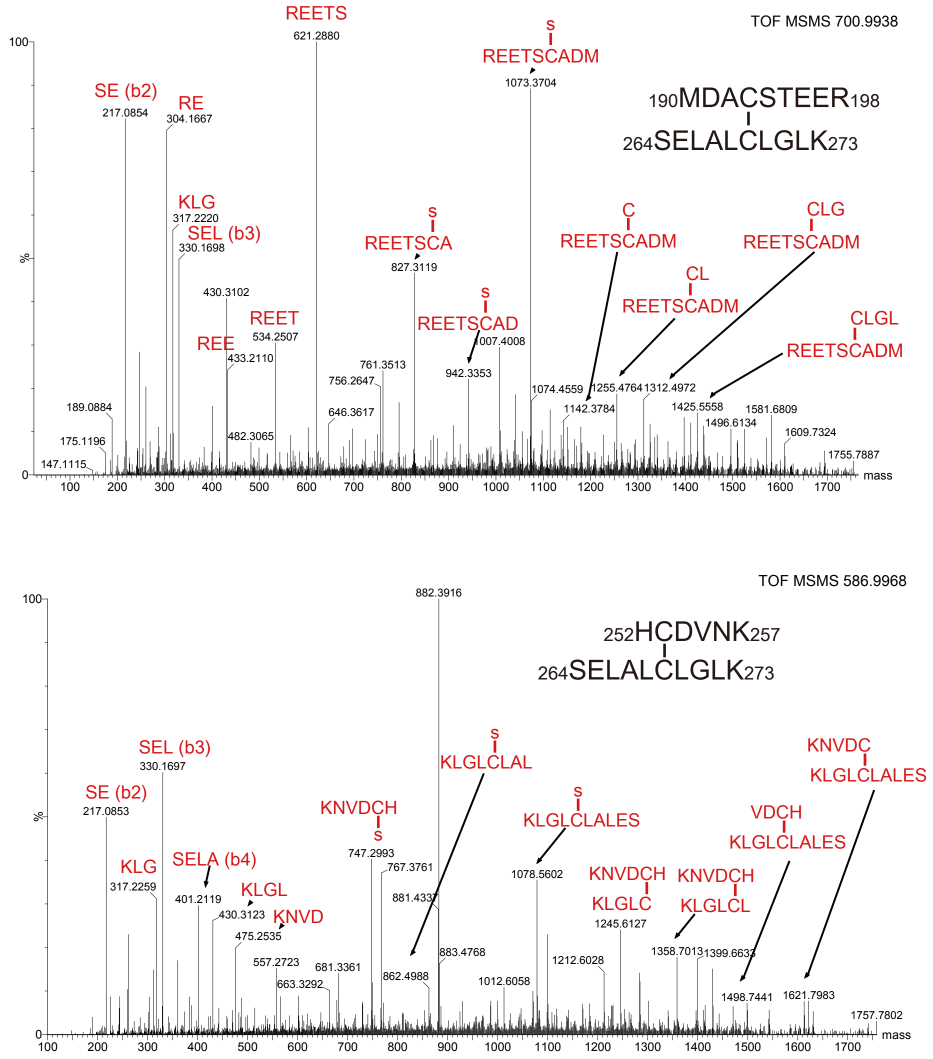

Figure S2

A

	Species	C193	C253	C269
MAMMALS	Humans	C	C	C
	Pan troglodytes (chimpanzee)	C	C	C
	Pan paniscus (bonobo)	C	C	C
	Gorilla gorilla gorilla (western lowland gorilla)	C	C	C
	Pongo abelii (Sumatran orangutan)	C	C	C
	Nomascus leucogenys (northern white-cheeked gibbon)	C	C	C
	Macaca mulatta (rhesus monkey)	C	C	C
	Macaca fascicularis (crab-eating macaque)	C	C	C
	Callithrix jacchus (white-tufted-ear marmoset)	C	C	C
	Mus musculus (mouse)	S	C	C
	Rattus norvegicus (rat)	S	C	C
	Cricetulus griseus (Chinese hamster)	S	C	C
	Nannospalax galili (Upper Galilee mountains blind mole rat)	C	C	C
	Heterocephalus glaber (naked mole rat)	S	C	C
	Oryctolagus cuniculus (rabbit)	C	C	C
	Tupaia chinensis (Chinese tree shrew)	C	C	C
	Canis familiaris (dog)	C	C	C
	Ailuropoda melanoleuca (giant panda)	C	C	C
	Ursus maritimus (polar bear)	C	C	C
	Felis catus (domestic cat)	C	C	C
	Panthera tigris altaica (Amur tiger)	C	C	C
	Bos taurus (cow)	C	C	C
	Bos mutus (wild yak)	C	C	C
	Pantholops hodgsonii (chiru)	C	C	C
	Capra hircus (goat)	C	C	C
	Ovis aries (sheep)	C	C	C
	Sus scrofa (pig)	C	C	C
	Camelus ferus (Wild Bactrian camel)	C	C	C
	Balaenoptera acutorostrata scammoni (minke whale)	C	C	C
	Lipotes vexillifer (Yangtze River dolphin)	C	C	C
	Equus caballus (horse)	C	C	C
	Myotis brandtii (Brandt's bat)	NG	NG	NG
	Myotis davidii	NG	NG	NG
	Pteropus alecto (black flying fox)	C	C	C
	Monodelphis domestica (opossum)	C	C	C
	Sarcophilus harrisii (Tasmanian devil)	C	C	C
	Ornithorhynchus anatinus (platypus)	C	C	C
BIRDS	Gallus gallus (chicken)	C	C	C
	Meleagris gallopavo (turkey)	C	C	C
	Anas platyrhynchos (mallard)	C	C	C
	Taeniopygia guttata (zebra finch)	C	C	C
	Geospiza fortis (medium ground-finch)	S	C	C
	Ficedula albicollis (collared flycatcher)	C	C	C
	Pseudopodoces humilis (Tibetan ground-tit)	C	C	C
	Corvus cornix (hooded crow)	C	C	C
	Falco peregrinus (peregrine falcon)	C	C	C
	Falco cherrug (Saker falcon)	C	C	C
	Columba livia (rock pigeon)	C	C	C
REPTILES	Alligator sinensis (Chinese alligator)	C	C	C
	Alligator mississippiensis (American alligator)	C	C	C
	Pelodiscus sinensis (Chinese soft-shelled turtle)	C	C	C
	Chelonia mydas (green sea turtle)	C	C	C
	Anolis carolinensis (green anole)	C	C	C
	Python bivittatus (Burmese python)	S	C	C
AMPHIBIANS	Xenopus laevis (African clawed frog)	S	C	C
	Xenopus tropicalis (western clawed frog)	S	C	C
FISHES	Danio rerio (zebrafish)	S	C	C
	Takifugu rubripes (torafugu)	C	C	C
	Maylandia zebra (zebra mbuna)	C	C	C
	Oryzias latipes (Japanese medaka)	C	C	C
	Xiphophorus maculatus (southern platyfish)	C	C	C
	Latimeria chalumnae (coelacanth)	C	C	C
CARTILAGINOUS FISHES	Callorhinchus milii (elephant shark)	C	C	C
LANCELETS	Branchiostoma floridae (Florida lancelet)	K	V	C
ASCIDIANS	Ciona intestinalis (sea squirt)	G	C	C
ECHINODERMS	Strongylocentrotus purpuratus (purple sea urchin)	M	F	C

	Species	C193	C253	C269
INSECTS	Drosophila melanogaster (fruit fly)	NG	NG	NG
	Drosophila pseudoobscura pseudoobscura	NG	NG	NG
	Drosophila ananassae	NG	NG	NG
	Drosophila erecta	NG	NG	NG
	Drosophila persimilis	NG	NG	NG
	Drosophila sechellia	NG	NG	NG
	Drosophila simulans	NG	NG	NG
	Drosophila willistoni	NG	NG	NG
	Drosophila yakuba	NG	NG	NG
	Drosophila grimshawi	NG	NG	NG
	Drosophila mojavensis	NG	NG	NG
	Drosophila virilis	NG	NG	NG
	Musca domestica (house fly)	NG	NG	NG
	Anopheles gambiae (mosquito)	NG	NG	NG
	Aedes aegypti (yellow fever mosquito)	NG	NG	NG
	Culex quinquefasciatus (southern house mosquito)	NG	NG	NG
	Apis mellifera (honey bee)	NG	NG	NG
	Solenopsis invicta (red fire ant)	NG	NG	NG
	Acromyrmex echinatior (Panamanian leafcutter ant)	NG	NG	NG
	Harpegnathos saltator (Jerdon's jumping ant)	NG	NG	NG
	Camponotus floridanus (Florida carpenter ant)	NG	NG	NG
	Nasonia vitripennis (jewel wasp)	NG	NG	NG
	Tribolium castaneum (red flour beetle)	NG	NG	NG
	Bombyx mori (domestic silkworm)	NG	NG	NG
	Plutella xylostella (diamondback moth)	NG	NG	NG
	Acyrthosiphon pisum (pea aphid)	NG	NG	NG
	Pediculus humanus corporis (human body louse)	NG	NG	NG
MITES AND TICKS	Ixodes scapularis (black-legged tick)	NG	NG	NG
NEMATODES	Caenorhabditis elegans (nematode)	NG	NG	NG
	Caenorhabditis briggsae	NG	NG	NG
	Brugia malayi (filaria)	NG	NG	NG
	Loa loa (eye worm)	NG	NG	NG
	Trichinella spiralis	NG	NG	NG
ANNELIDS	Helobdella robusta	NG	NG	NG
MOLLUSKS	Lottia gigantea (owl limpet)	NG	NG	NG
	Crassostrea gigas (Pacific oyster)	NG	NG	NG
FLATWORMS	Schistosoma mansoni	NG	NG	NG
CNIDARIANS	Nematostella vectensis (sea anemone)	NG	NG	NG
	Hydra vulgaris	NG	NG	NG
PLACOZOANS	Trichoplax adhaerens	NG	NG	NG
PORIFERANS	Amphimedon queenslandica (sponge)	NG	NG	NG
		HIGH	HIGH	HIGH

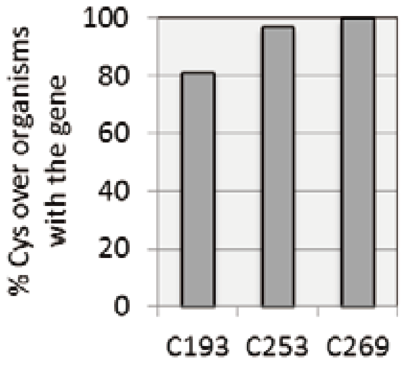

"NG" means there is no true ortholog (no gene) in that species

Figure S3

B

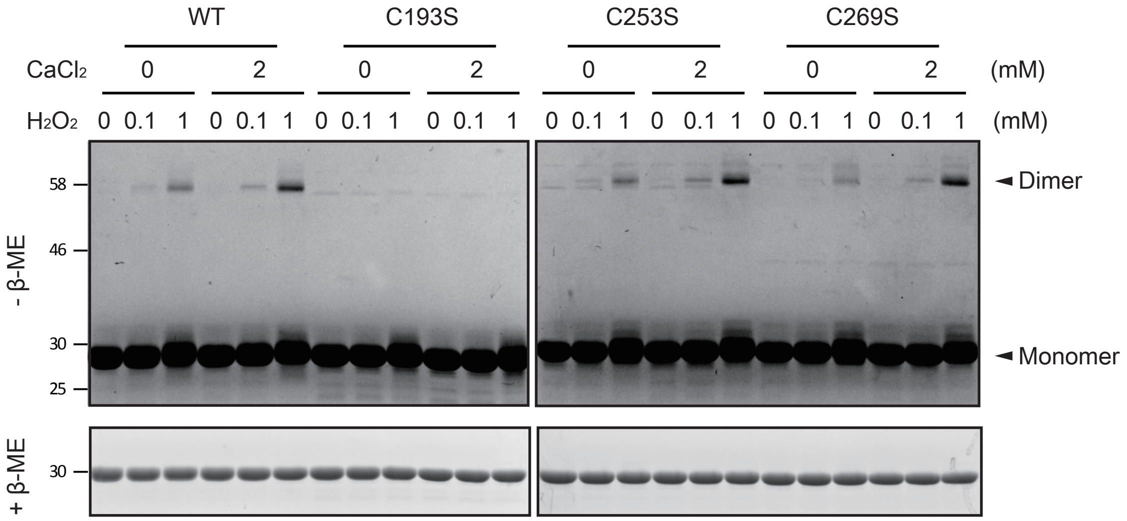

C

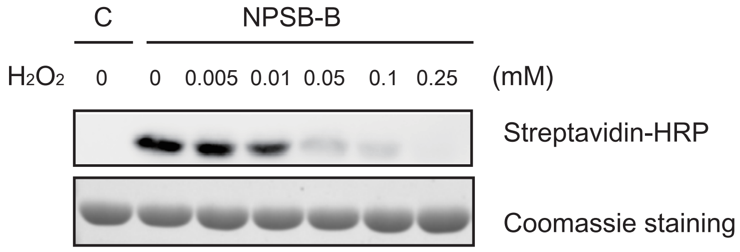

Figure S3 (continued)

Table S1 - Differential deuterium exchange rates of identified SCGN peptides in HDX-MS experiment.

Sequence	Start	End	Domain	Structure (Residue no.)	Differential deuterium exchange rate (%)					Mean
					10 sec	60 sec	300 sec	1800 sec	10800 sec	
DSSREPTLGRL	2	12			0.00	0.00	0.00	0.00	0.00	0.00
DSSREPTLGRLDAAGF	2	17	Domain I		6.25	3.13	0.00	0.00	0.00	1.88
WQVWQRF**DADE**	18	28		EF 1 (25-36)	18.18	18.18	18.18	9.09	4.46	13.62
WQVWQRF**DADEKGYIEE**	18	34			17.65	11.76	17.65	0.00	0.00	9.41
**YIEEKE**LDAFF	31	41			9.09	9.09	9.09	18.18	0.00	9.09
FLHMLMKLGTDD	41	52			0.00	0.00	0.00	0.00	0.00	0.00
FLHMLMKLGTDDTVM	41	55			0.00	0.00	0.00	0.00	0.00	0.00
LMKLGTDDTVM	45	55			0.00	0.00	0.00	0.00	0.00	0.00
LMKLGTDDTVMKANL	45	59			0.00	0.00	0.00	0.00	0.00	0.00
KANLHKVKQQF	56	66			9.09	0.00	-9.09	0.00	0.00	0.00
MTTQ**DASKDGRIRMKE**LAGMF	67	87		EF 2 (71-82)	4.76	7.14	4.76	4.76	4.76	5.24
**KE**LAGMF	81	87			14.29	7.14	14.29	0.00	0.00	7.14
LLFRRENPLDSSVEF	96	110	Linker 1		10.00	6.67	6.67	0.00	0.00	4.67
LFRRENPLDSSVEF	97	110			3.57	10.71	3.57	0.00	0.00	3.57
FRRENPLDSSVEF	98	110			0.00	0.00	0.00	0.00	0.00	0.00
MQIWRKY**DADSSG**	111	123	Domain II	EF 3 (118-129)	-7.69	-3.85	0.00	0.00	0.00	-2.31
WRKY**DADSSGF**	114	124			-9.09	-9.09	0.00	0.00	-18.18	-7.27
**ISAAE**LRNFLRD	125	136			0.00	0.00	0.00	0.00	0.00	0.00
**AE**LRNFLRDLFLHHKKAISE	128	147			-2.50	-2.50	-5.00	0.00	-7.50	-3.50
LRNFLRDLFLHHKKAISE	130	147			-5.56	0.00	0.00	0.00	-2.78	-1.67
AKLEEYTGTM	148	157			0.00	-15.00	-10.00	-10.00	0.00	-7.00
EEYTGTM	151	157		EF 4 (162-173)	-14.29	0.00	7.14	-14.29	0.00	-4.29
MKIF**DRNKDGRLDLND**L	158	174			0.00	0.00	0.00	-8.82	-5.88	-2.94
**DLND**LARILALQENF	170	184			3.33	6.67	0.00	0.00	0.00	2.00
ARILALQENFLL	175	186			-8.33	-4.17	4.17	4.17	4.17	0.00
QENFLLQFKMDA**C**ST	185	195	Linker 2		9.09	9.09	9.09	0.00	0.00	5.45
EERKRDFEKIFA	196	207	Domain III		0.00	0.00	-8.33	0.00	0.00	-1.67
AYY**DVSKTGALEGPE**VDG	207	224		EF 5 (210-221)	0.00	-5.56	-16.67	-5.56	0.00	-5.56
AYY**DVSKTGALEGPE**VDGF	207	225			-7.89	-10.53	-21.05	-10.53	0.00	-10.00
YY**DVSKTGALEGPE**VDG	208	224			-5.88	-5.88	-17.65	-5.88	0.00	-7.06
Y**DVSKTGALEGPE**VDG	209	224			-6.25	-6.25	-18.75	-6.25	0.00	-7.50
ELVQPSISGVDLDKF	231	245			-13.33	0.00	0.00	0.00	0.00	-2.67
ELVQPSISGVDLDKFREIL	231	249			-5.26	0.00	-10.53	-5.26	0.00	-4.21
LVQPSISGVDLDKF	232	245			-10.71	0.00	-7.14	0.00	0.00	-3.57
PSISGVDLDKFREIL	235	249			-6.67	0.00	-6.67	-6.67	-6.67	-5.33
LRH**CDVNKDGKIQKSE**L	250	266		EF 6 (254-265)	-11.76	-11.76	-17.65	-17.65	-11.76	-14.12
**C**LGLKIN	269	276			0.00	12.50	0.00	0.00	0.00	2.50

Table S2 - Amino acids sequences of the six EF-hand loops of SCGN.
The amino acids with low occurrence (less than 5%) at each position in known EF-loops are underlined (Gifford, Walsh et al., 2007).

EF-loop position	1	2	3	4	5	6	7	8	9	10	11	12
	**+X (D)**		**+Y (D,N)**		**+Z (D,S,N)**				**-X (D,S,T,E ,N,G,Q)**			**-Z (E,D)**
EF1 (D25-E36)	D	A	D	E	K	G	Y	I	E	E	K	E
EF2 (D71-E82)	D	A	S	K	D	G	R	I	R	M	K	E
EF3 (D118-E129)	D	A	D	S	S	G	F	I	S	A	A	E
EF4 (D162-D173)	D	R	N	K	D	G	R	L	D	L	N	D
EF5 (D210-E221)	D	V	S	K	T	G	A	L	E	G	P	E
EF6 (D254-E265)	D	V	N	K	D	G	K	I	Q	K	S	E

A

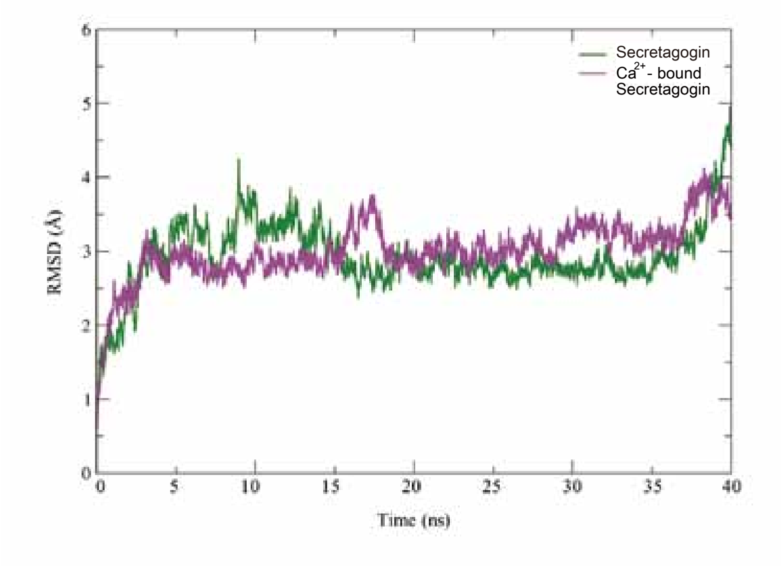

Secretagogin
Ca^{2+} - bound Secretagogin
RMSD (Å)
Time (ns)
0
1
2
3
4
5
6
0
5
10
15
20
25
30
35
40

B

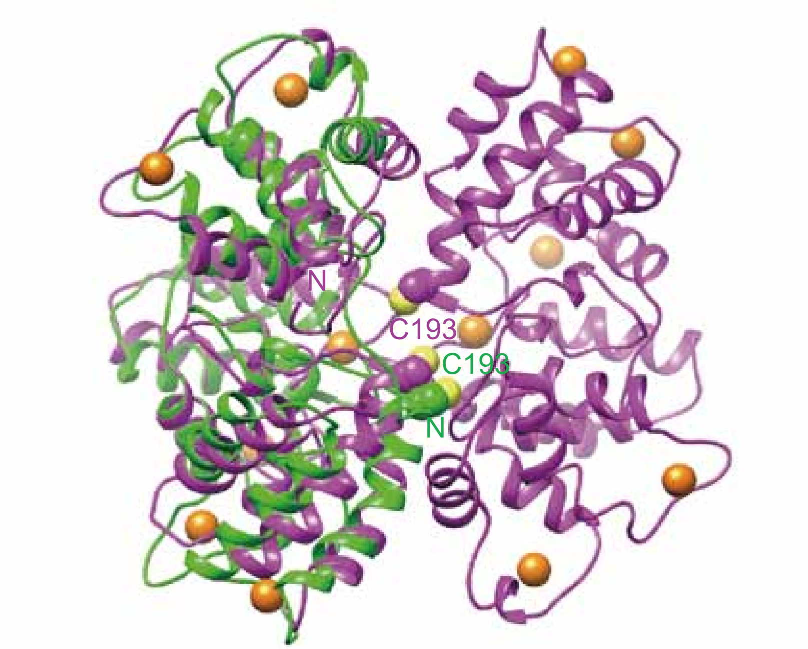

N
C193
C193
N

Figure S4

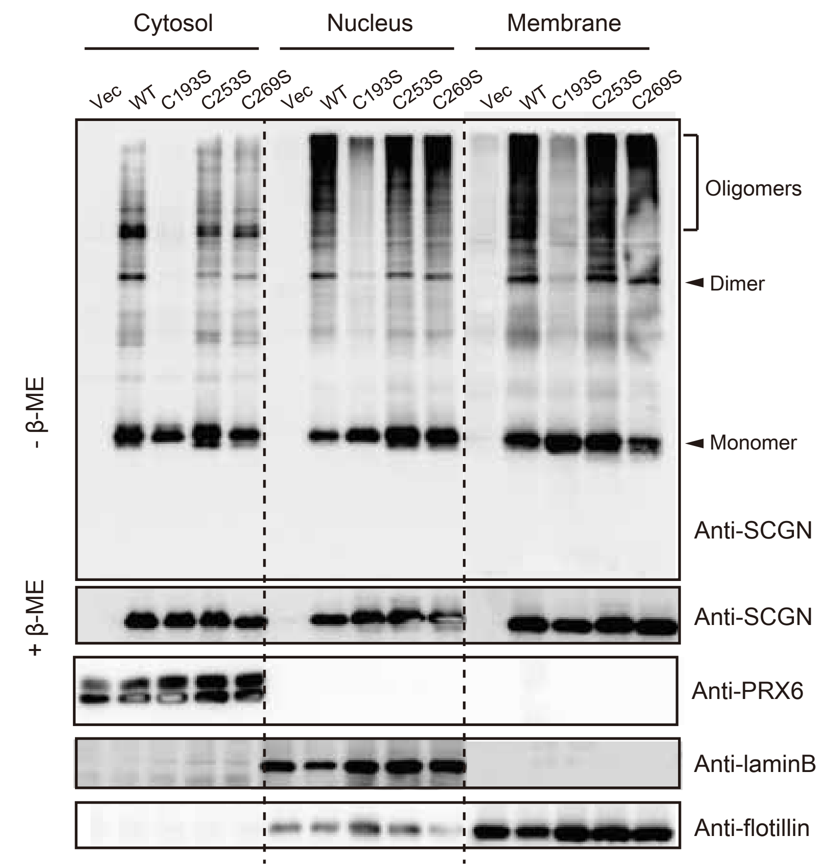
Cytosol
Nucleus
Membrane
Vec
WT
C193S
C253S
C269S
Vec
WT
C193S
C253S
C269S
Vec
WT
C193S
C253S
C269S
Oligomers
Dimer
Monomer
- β-ME
Anti-SCGN
+ β-ME
Anti-SCGN
Anti-PRX6
Anti-laminB
Anti-flotillin

Figure S5

Figure 1A

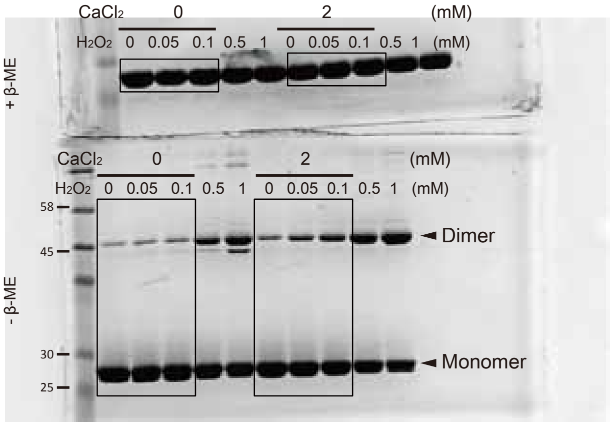

Figure 1B

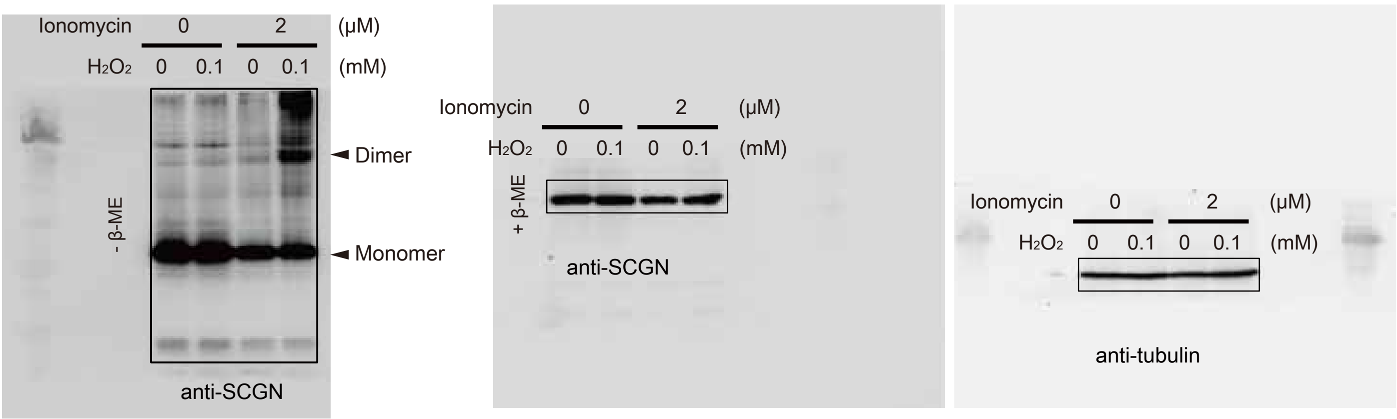

Figure 1D

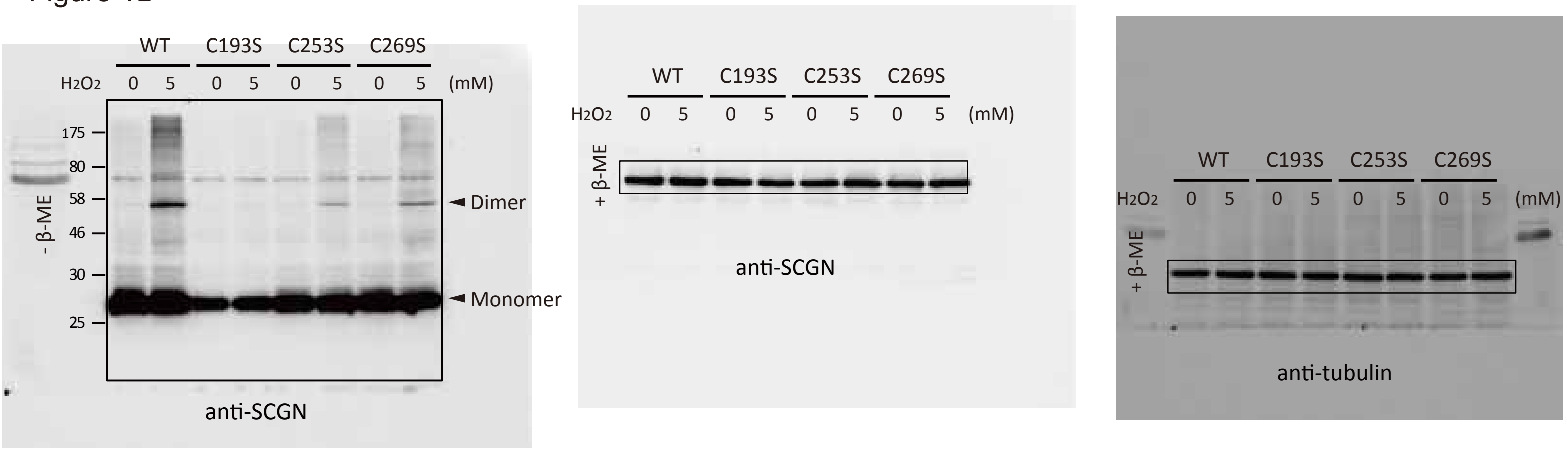

Figure 1E

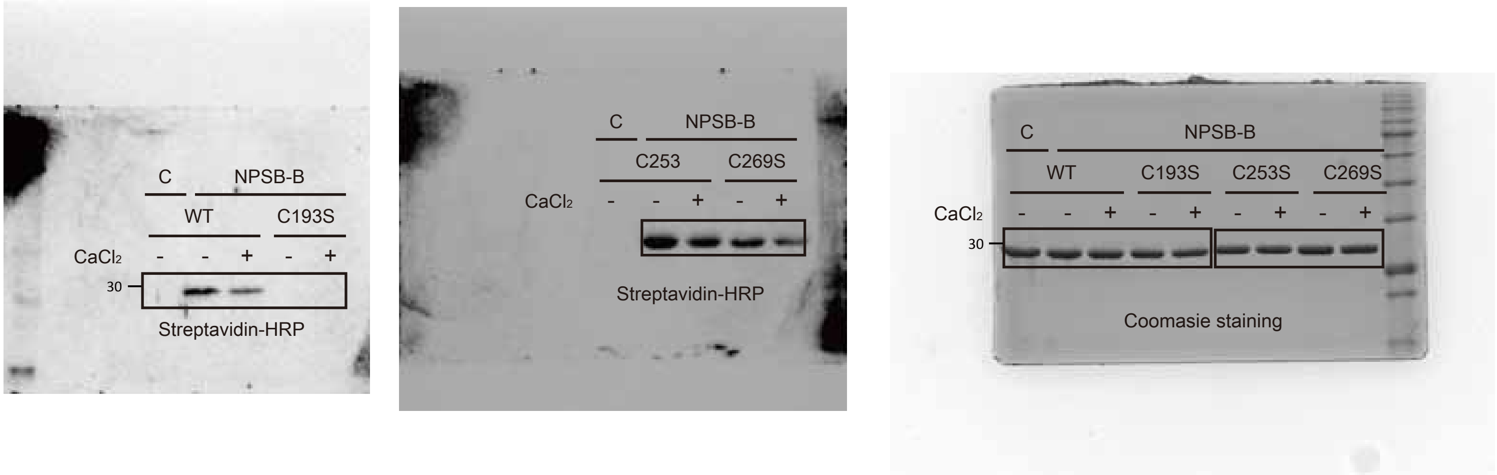

Figure S6

Figure 4A

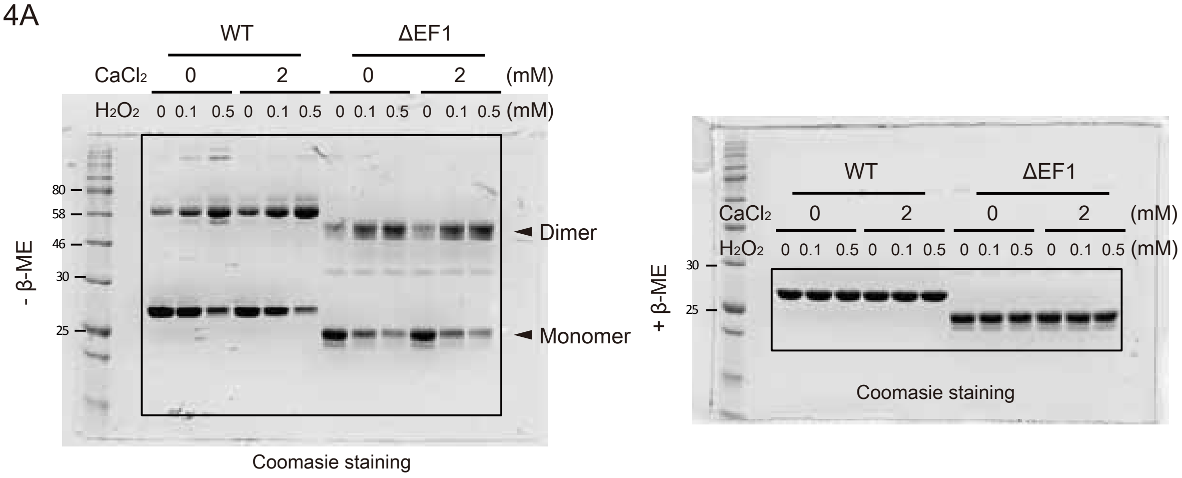

Figure 4B

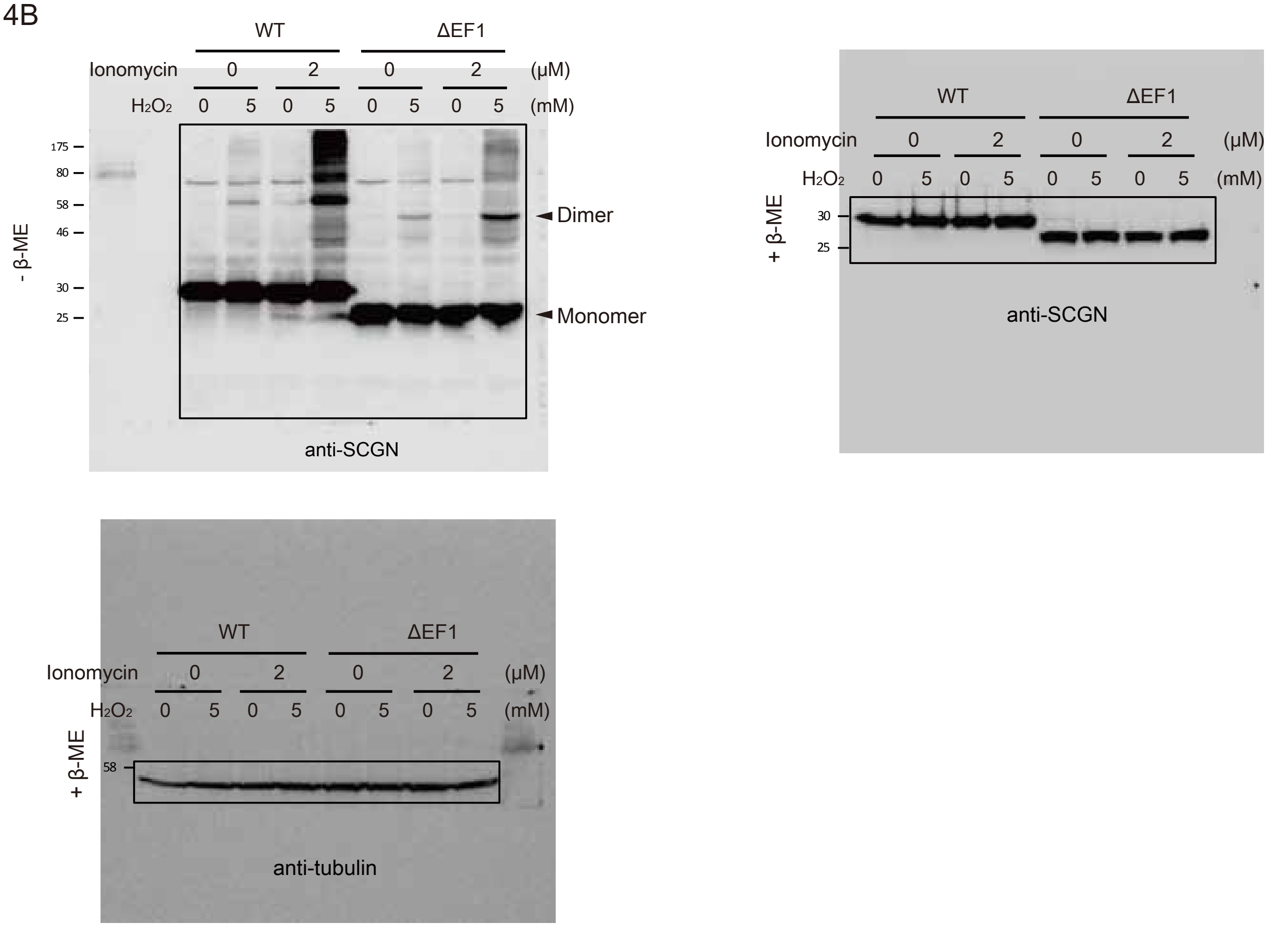

Figure 4C

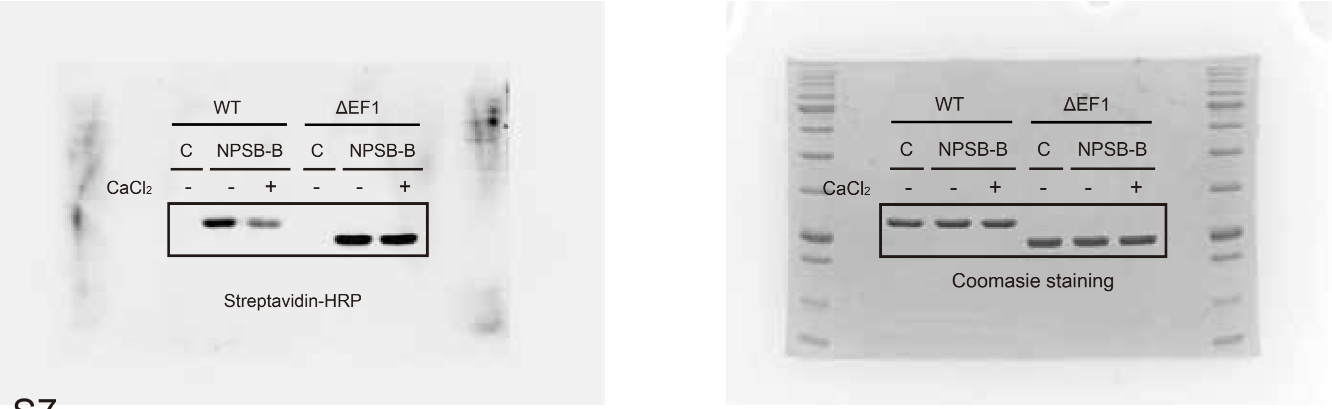

Figure S7

Figure 5A

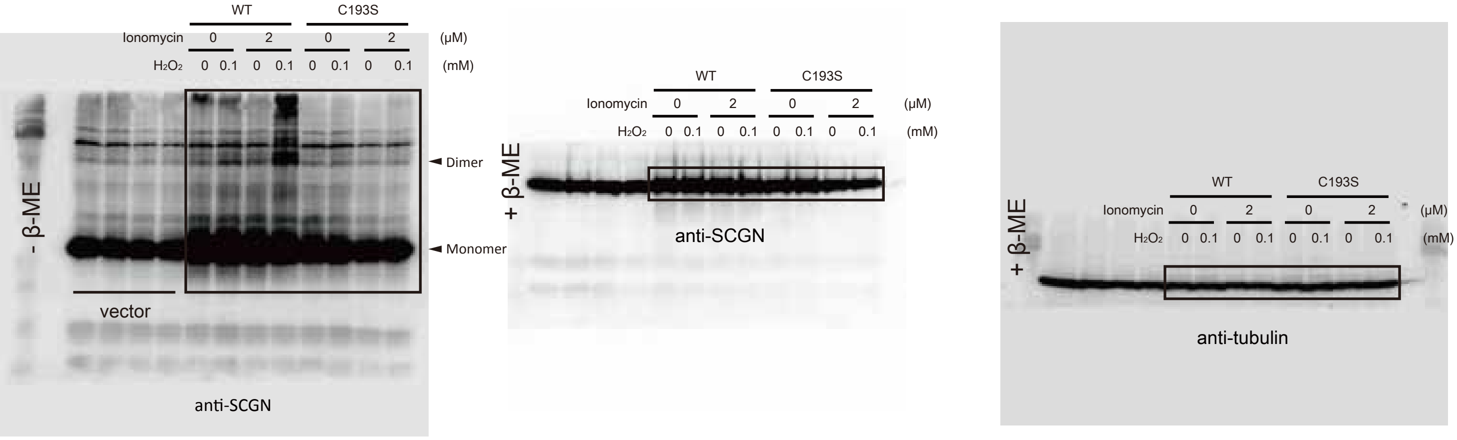

Figure 5B

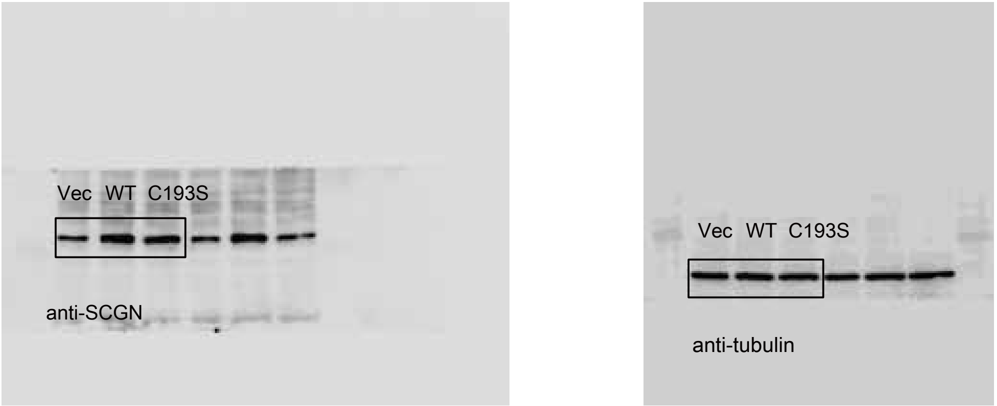

Figure 5C

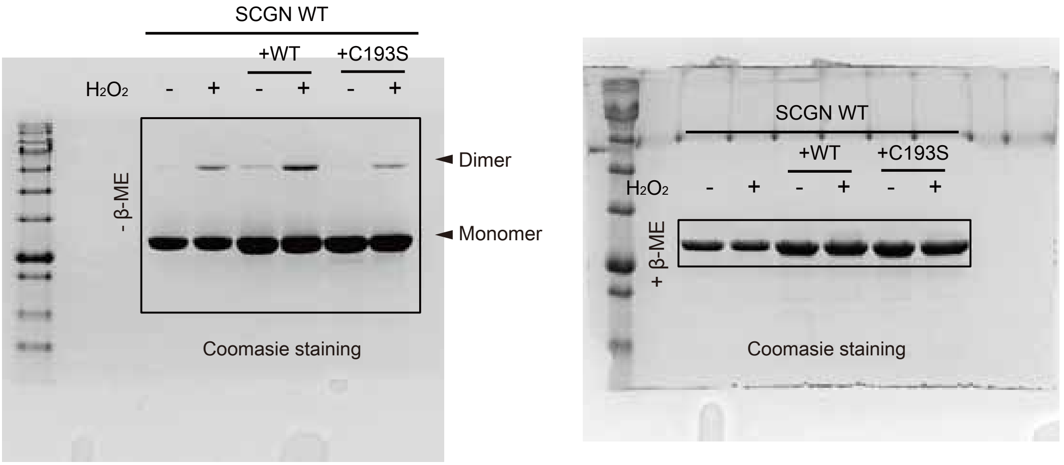

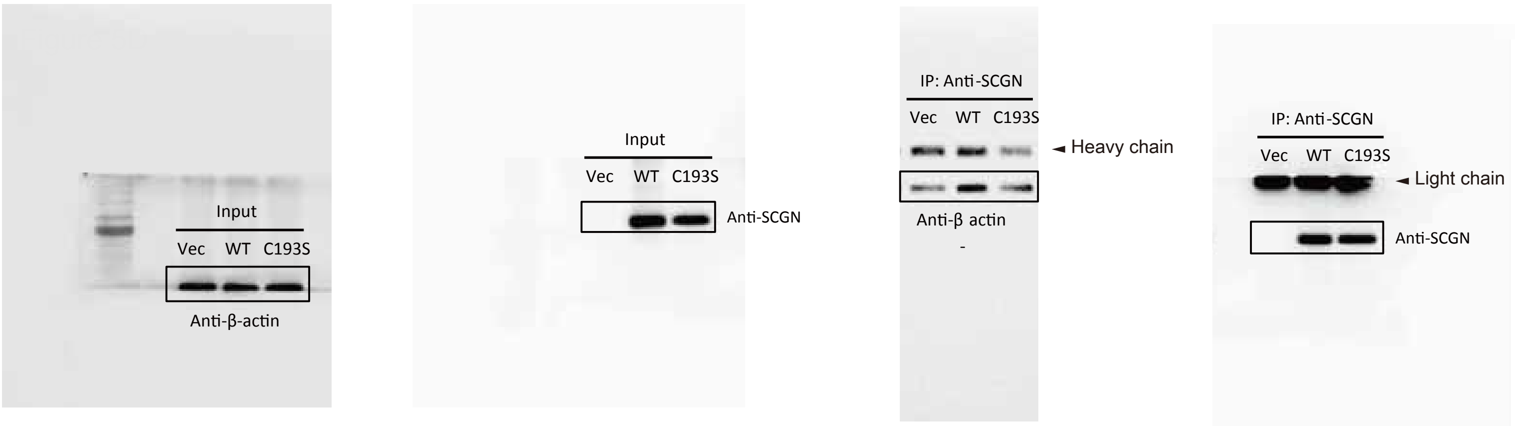

Figure S8
